# Acetylation-regulated DUSP1 deficiency contributes to renal fibrosis progression

**DOI:** 10.7150/thno.108992

**Published:** 2025-03-03

**Authors:** Shaobo Wang, Bo Zhang, Yaqin Wang, Qigang Lan, Liangjing Lv, Tangli Xiao, Yan Li, Mengying Yao, Jun Zhang, Cheng Wang, Yinghui Huang, Jinghong Zhao, Jiachuan Xiong

**Affiliations:** 1 Department of Nephrology, Key Laboratory for the Prevention and Treatment of Kidney Disease of Chongqing, Chongqing Clinical Research Center of Kidney and Urology Diseases, Xinqiao Hospital, Army Medical University (Third Military Medical University), Chongqing 400037, China.; 2 State Key Laboratory of Trauma, Burns and Combined Injury, Institute of Combined Injury, Chongqing Engineering Research Center for Nanomedicine, College of Preventive Medicine, Army Medical University (Third Military Medical University), Chongqing, China.

**Keywords:** kidney fibrosis, Acetylation, Dual specificity phosphatase 1, Smad, dephosphorylation

## Abstract

**Rationale:** The irreversible damage of renal fibrosis has been widely recognized as a critical factor in the progression of chronic kidney disease (CKD) to end-stage kidney failure. This necessitates investigation into its precise mechanisms. Dual-specificity phosphatase 1 (DUSP1), a regulator of mitogen-activated protein kinase (MAPK) pathways, is linked to diseases such as cancer and immune disorders, but its role in renal fibrosis is unclear. This study aimed to clarify the role of DUSP1 in renal fibrosis, identify the intrinsic mechanisms involved, and provide a theoretical basis for the clinical translation of a new target for renal fibrosis treatment.

**Methods:** We characterized DUSP1 expression in kidney tissues from unilateral ureteral obstruction (UUO) mice and patients with CKD using histological analysis. We established a UUO-induced renal fibrosis model using DUSP1 knockout mice. The role and mechanism of DUSP1-mediated inhibition of renal fibrosis was evaluated both *in vivo* and *in vitro*. Finally, we performed virus-mediated gene transfer, RNA-Seq, immunohistochemistry, western blotting, and qPCR to further analyze our findings.

**Results:** We found that DUSP1, a crucial dephosphorylating enzyme, was remarkably reduced in renal tubular epithelial cells (RTECs) in mice and patients with CKD. This reduction was inversely correlated with kidney function and severity of renal fibrosis. DUSP1 deficiency exacerbated UUO-induced renal fibrosis in mice, whereas overexpression of DUSP 1 reduces fibrogenesis in human renal tubular epithelial (HK-2) cells treated with transforming growth factor-β1 *in vitro*. Mechanistically, deletion of DUSP1 promotes the nuclear translocation of Smad3, a crucial mediator of renal fibrosis, primarily through dephosphorylation at its 423/425 residue. Interestingly, we observed that DUSP1 is primarily regulated by acetylation modification, which is accompanied by an increased expression of histone deacetylase 1 (HDAC1) under UUO conditions. Furthermore, HDAC1 inhibition reversed the decrease in DUSP1 and the dephosphorylation of Smad3 in RTECs. Finally, the use of HDAC1 inhibitors or adeno-associated virus-mediated DUSP1 overexpression in RTECs significantly ameliorated UUO-induced renal injury and fibrosis.

**Conclusion:** These results demonstrate that DUSP1 deficiency accelerates renal fibrosis through Smad3 nuclear translocation, modulated by HDAC1-driven acetylation. HDAC1 inhibition or DUSP1 overexpression significantly alleviated renal damage, highlighting DUSP1's therapeutic potential in combating CKD progression.

## Introduction

Chronic kidney disease (CKD), which is characterized by an excessive buildup of extracellular matrix (ECM) proteins and eventually results in end-stage kidney disease (ESKD), frequently follows this pathway and is characterized by renal interstitial fibrosis (TIF) 1, 2. Until now, the etiology and pathogenesis of renal fibrosis remain unclear, and effective preventive and treatment strategies are still lacking. Renal tubular epithelial cells (RTECs) are the main components of the renal parenchyma, and extensive evidence has shown that they are the initial cells of fibrosis 3. The RTECs are susceptible to injury as a consequence of ischemia, contrast agents, toxins, and other factors. In contrast, injured RTECs exhibit cell cycle arrest, metabolic reprogramming, and release of pro-inflammatory proteins, all of which enhance myofibroblast activation 4, 5. Furthermore, persistent inflammation promotes renal fibrosis and contributes to the development of diseases 6. Evidence suggests that injured RTECs release pro-fibrotic factors during the activation stage of the disease. In response to these factors, myofibroblasts produce an extracellular matrix that results in the progression of fibrosis 7, 8. Dysregulation of the TGF-β/Smad3 signaling pathway has been implicated in various diseases, including cancer, fibrosis, and immune disorders 9-12. Studies have confirmed the activation of TGF-β/Smad3, a signaling pathway that contributes to fibrogenic processes in RTECs 13, 14. Furthermore, the persistent activation of cytokines and degradation of the basal membrane of renal tubules by local proteases during inflammation might trigger the transdifferentiation of RTECs into mesenchymal cells, disrupting normal cell repair and leading to fibrosis 15. Moreover, amount of evidence has shown that Smad3 activation is involved in renal fibrosis 16, 17. Genetic or pharmacological inhibition of Smad3 has been shown to attenuate renal fibrosis in several animal models of CKD, including diabetic nephropathy, glomerulonephritis, and obstructive nephropathy 17. Thus, targeting the TGF-β/Smad3 signaling pathway and its crosstalk with other signaling pathways represents a promising therapeutic approach for treating renal fibrosis.

Dual-specificity phosphatase 1 (DUSP1), a key member of the nuclear mitogen-activated protein kinase (MAPK)-specific phosphatase family, functions as a negative regulator of various signaling pathways by dephosphorylating specific tyrosine and threonine residues on target proteins 18. It has been implicated in a variety of cellular processes, including cell proliferation, differentiation, and apoptosis, as well as in disease states, such as cancer and immune disorders 19, 20. For example, DUSP1 expression is often altered in cancer cells and has been shown to regulate tumor growth and invasion by modulating the activity of oncogenic signaling pathways such as MAPK and JNK 21. In addition, DUSP1 has been identified as a potential therapeutic target for autoimmune diseases such as multiple sclerosis, which suppresses the activation of immune cells and the release of pro-inflammatory cytokines 22. However, the role of DUSP1 in kidney disease is largely unknown, especially in the pathogenesis of renal fibrosis.

In the present study, we provided compelling data to substantiate that DUSP1 plays a pivotal role in renal fibrosis. Our findings indicate that DUSP1 expression is significantly decreased in the kidneys of both animal models and CKD patients. Further studies have revealed that the overexpression of DUSP1 protects against renal fibrosis by regulating the dephosphorylation of Smad3, a crucial process in the development of kidney fibrosis. These exciting findings have identified DUSP1 as a major player in the pathogenesis of kidney fibrogenesis and have provided a promising avenue for future research.

## Materials and methods

### Human tissues

Kidney tissues from patients with CKD were obtained using a biopsy procedure. A total of 21 CKD patients with various etiologies were included in this study (Table S1). The study was conducted according to ethical guidelines and was approved by the Army Medical University (NO: 2018-006-02), and informed consent was obtained from all patients with CKD. A paraffin embedding technique was employed to prepare tissue samples for analysis. The tissues were embedded in paraffin wax and cutting them into sections with a thickness of 3 mm. The sections were then subjected to deparaffinization, hydration, blocking, and incubation with specific antibodies (Anti-DUSP1, #48625, CST, 1:100. Anti-HDAC1, ab109411, Abcam, 1:100). After incubation, the tissue sections were stained to visualize the presence and distribution of target molecules. To capture the staining patterns, the stained tissue sections were scanned using advanced imaging technology.

### Mouse model of UUO

The UUO model was established as previously described 23. Briefly, mice were anesthetized by intraperitoneal injection of pentobarbital sodium. A surgical incision was made in the flank, and the left ureter was tied using sterile 4-0 silk sutures at two points. Both obstructed and contralateral kidneys were harvested on day 14 post-ureteral ligation for further analysis. At least 6 pairs of mice were used in each assay. The mice were euthanized after anesthesia. Kidneys were removed and prepared for histological evaluation and protein extraction or stored at -80℃. All animal use and adenoviral protocols were approved by the Institutional Animal Care and Use Committee of the Army Medical University and were conducted in accordance with the animal care guidelines of the Army Medical University.

### Scr and BUN measurements

Blood samples were collected and centrifuged at 3000 rpm for 15 min. Upper serum samples were collected and analyzed. Serum creatinine (Scr) and blood urea nitrogen (BUN) levels were determined using a commercial kit (Nanjing Jiancheng Bioengineering Institute, CHN) following the manufacturer's instructions, as previously reported 23.

### Histology evaluation

Renal histology was evaluated by blind examination following H&E staining. To ensure a comprehensive analysis, ten fields per section were randomly selected from each tissue section at a magnification of ×200.

### Cell culture and treatment

Immortalized human renal proximal tubular epithelial cells (HK-2 cells) were obtained from the Chinese National Infrastructure of Cell Line Resource (Beijing, China). All cell lines used in this study were confirmed free of mycoplasma contamination. HK-2 cells were cultured in DMEM-F12 medium supplemented with 10%fetal bovine serum (FBS; #10099141 C, Gibco, USA). For TGF-β (Pepro Tech, 100-21-10) treatment, HK-2 cells were treated with TGF-β for 24 h. To establish DUSP1 stable overexpression cells, DUSP1-specific plasmids were purchased from Tsingke Biotechnology (Beijing, China) and then introduced into HK-2 cells when they reached 60-70% confluence. The efficiency of DUSP1 overexpression was confirmed using immunoblotting. To generate DUSP1 or Hdac1 stable knockdown cells, DUSP1 or HDAC1 specific siRNA were purchased from Tsingke Biotechnology (Beijing, China). The knockdown efficiencies of DUSP1 and Hdac1 were confirmed by immunoblotting. The information for all the siRNA sequences used is listed in Table S2.

### Real-time fluorescence quantitative polymerase chain reaction (RT-qPCR)

Total RNA was extracted using an RNA isolation kit (R0027, Beyotime Biotechnology, China) according to the manufacturer's instructions. First-strand cDNA was obtained by reverse transcription using RT Master Mix for qPCR II (TSK314M, Tsingke Biotechnology, China). Levels of DUSP1, Fibronectin, Collagen I, α-SMA, Hdac1, Hdac2, Hdac3, Hdac4, Hdac5, Hdac6, Hdac7, Hdac8, Hdac9, Hdac10, and Hdac11 mRNA were measured using SYBR Green qPCR Master mix (TSE204, Tsingke Biotechnology, China) and normalized to the levels of β-Actin mRNA. All primers were commercially synthesized (Sangon Biotech, China), and their sequences are summarized in Table S3.

### Immunoblotting analysis

The cells were lysed in RIPA lysis buffer (P0013B, Beyotime Biotechnology, China) containing a protease inhibitor cocktail (#11873580001, Roche, Switzerland) and phosphatase inhibitors (#4906845001, Roche, Switzerland). Equal amounts of protein (30 μg) were separated by 8-12% SDS-PAGE and transferred to a PVDF membrane (#03010040001, MERCK, USA). The primary antibodies were diluted in a Primary Antibody Dilution Buffer (P0023A, Beyotime Biotechnology, China). Membranes were incubated with primary antibodies overnight at 4°C. After thorough washing, HRP-labeled secondary antibodies (#7074, #7076, CST, USA, 1:3000) were added for 1h at 37°C. The ECL reagent was used to visualize the proteins (ab133406, Abcam, USA). Information for all antibodies used is listed in Table S4.

### Immunofluorescence staining and immunohistochemistry staining

For immunohistochemical staining, the sections were treated with sodium citrate at 100°C for 15 min. After blocking with Immunol Staining Blocking Buffer (P0102, Beyotime Biotechnology, China) for 10 min, the sections were incubated with the primary antibody at 4°C overnight and washed with TBS three times. They were then incubated with an anti-rabbit/secondary antibody (PV-6002/PV-9001, ZSBGBIO, China). Sections were washed three times with TBS and stained with DAB solution (ZLI-9019, ZSBGBIO, China) at room temperature for 1-5 min. Slides were photographed using a confocal microscope (ZEISS, LSM780) and analyzed using ZEN 2012 software (version 1.1.1.0, Carl Zeiss Microscopy GmbH).

### Construction of DUSP1 mutant plasmids

The cDNAs of DUSP1 and SMAD3 were subcloned into pcDNA3.0-FLAG and pcDNA3.0-HIS vectors, respectively. Mutagenic primers targeting amino acids 258 and 259 of the DUSP1 gene were designed and point mutations were introduced using the Mut Express II Fast Mutagenesis Kit V2 (Vazyme, C214). The forward primer sequence was 5'-TTTGTCCACcgactaGCAGGCATTTCCCGGTCA-3', and the reverse primer sequence was 5'-TGCtagtcgGTGGACAAACACCCTTCCTCCAGC-3'. Subsequently, both mutant and wild-type DUSP1 plasmids were transfected into 293T cells and subsequent experiments were conducted.

### Chromatin immunoprecipitation and Co-immunoprecipitation assays

The ChIP experiment was conducted using a SimpleChIP Enzymatic Chromatin IP Kit (#56383, CST, USA) following the manufacturer's instructions. Co-immunoprecipitated DNA was extracted, purified after ChIP, and used as a template for subsequent PCR. Information for all ChIP primer sequences of the oligos used is summarized in Table S5. *In vitro* co-immunoprecipitation assays were performed as follows: cells were washed with chilled TBS and lysed in 200 µL of RIPA buffer (P0013B, Beyotime Biotechnology, China) containing a cocktail of protease inhibitors (#7012, CST, USA) and phosphatase inhibitors (#5870, CST, USA). Co-immunoprecipitation was performed using the Pierce Classic Magnetic IP/Co-IP Kit (88804, Thermo Fisher Scientific, USA) according to the manufacturer's instructions.

### Statistical analysis

Qualitative data, including tissue images, represented findings from at least three experiments. Quantitative data were reported as mean ± standard deviation (SD). Statistical analysis was performed using the GraphPad Prism 8 software (GraphPad Software Inc., La Jolla, CA, USA). Multiple groups were compared using analysis of variance (ANOVA), followed by Tukey's post-hoc test. Statistical differences between two groups were determined using two-tailed unpaired or paired t-tests. Statistical significance was set at P < 0.05.

## Results

### DUSP1 was downregulated in renal tubular epithelial cells in UUO mice and patients with CKD

**Figure 1:**
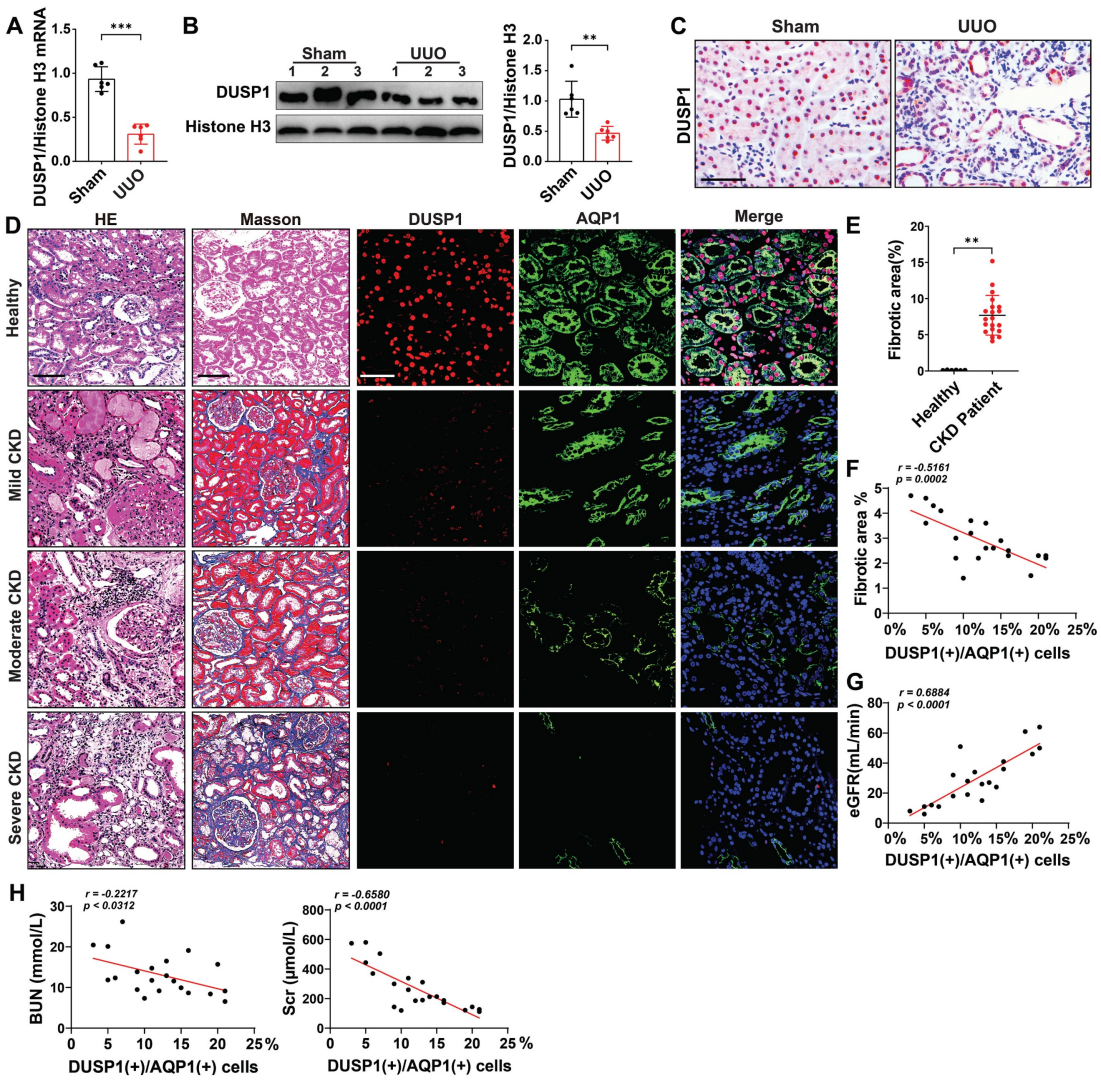
** DUSP1 is downregulated in renal tubular epithelial cells of UUO mice and patients with CKD.** (A) Relative mRNA and (B) protein expression levels of DUSP1 in the kidneys of sham and UUO mice (n = 6). (C) Representative images of immunohistochemical staining and quantitative analysis of DUSP1 expression. Scale bar, 100 μm. (D) H&E, Masson, and DUSP1 staining images of kidney biopsies from healthy controls and patients with CKD. Left two columns: Scale bars, 100 µm; right three columns: Scale bars, 50 µm. (E-H) Correlation analysis of the percentage of DUSP1 in renal tubules with renal fibrosis, eGFR, BUN, and Scr in individuals with chronic kidney disease (n = 21). *p < 0.05, **p < 0.01, and ***p < 0.001, n.s., not significant, student t test (A, B and E). Pearson's correlation coefficients (F, G, H).

Our initial investigation, using the online kidney single-cell sequencing database (https://humphreyslab.com/SingleCell/), revealed that DUSP1 expression was elevated in RTECs, particularly in the s1-s2 segment (Figure S1A-B). However, in a mouse model of unilateral ureteral obstruction (UUO), the expression of DUSP1 was significantly lower than that of the other members of the DUSP family (Figure S1C-D). To extend these findings, we analyzed DUSP1 expression in human kidneys using the Nephroseq database (http://www.nephroseq.org). A significant reduction in DUSP1 mRNA levels was observed in CKD tissues compared to normal kidney tissues (Figure S1E). This pattern was further validated in the UUO mouse model, where both mRNA and protein levels of DUSP1 were significantly reduced compared to those in sham controls (Figure 1A-C). The expression of DUSP1 in renal tissue was also continuously reduced in the UUO model on days 3 and 7 (Figure S1F). To explore the clinical relevance of these observations, we assessed DUSP1 expression in kidney biopsies of patients with CKD. Immunohistochemical staining revealed a marked reduction in DUSP1 expression in fibrotic kidneys compared to normal kidneys (Figure 1D-E, Figure S1G). More importantly, DUSP1 expression was inversely correlated with the severity of kidney fibrosis and positively correlated with the estimated glomerular filtration rate (eGFR), as well as a negative relationship with serum creatine and blood nitrogen (Figure 1F-H). In summary, these findings underscore the significant role of DUSP1 in kidney function and its involvement in fibrosis.

### DUSP1 deficiency promoted renal fibrosis in UUO mice

**Figure 2:**
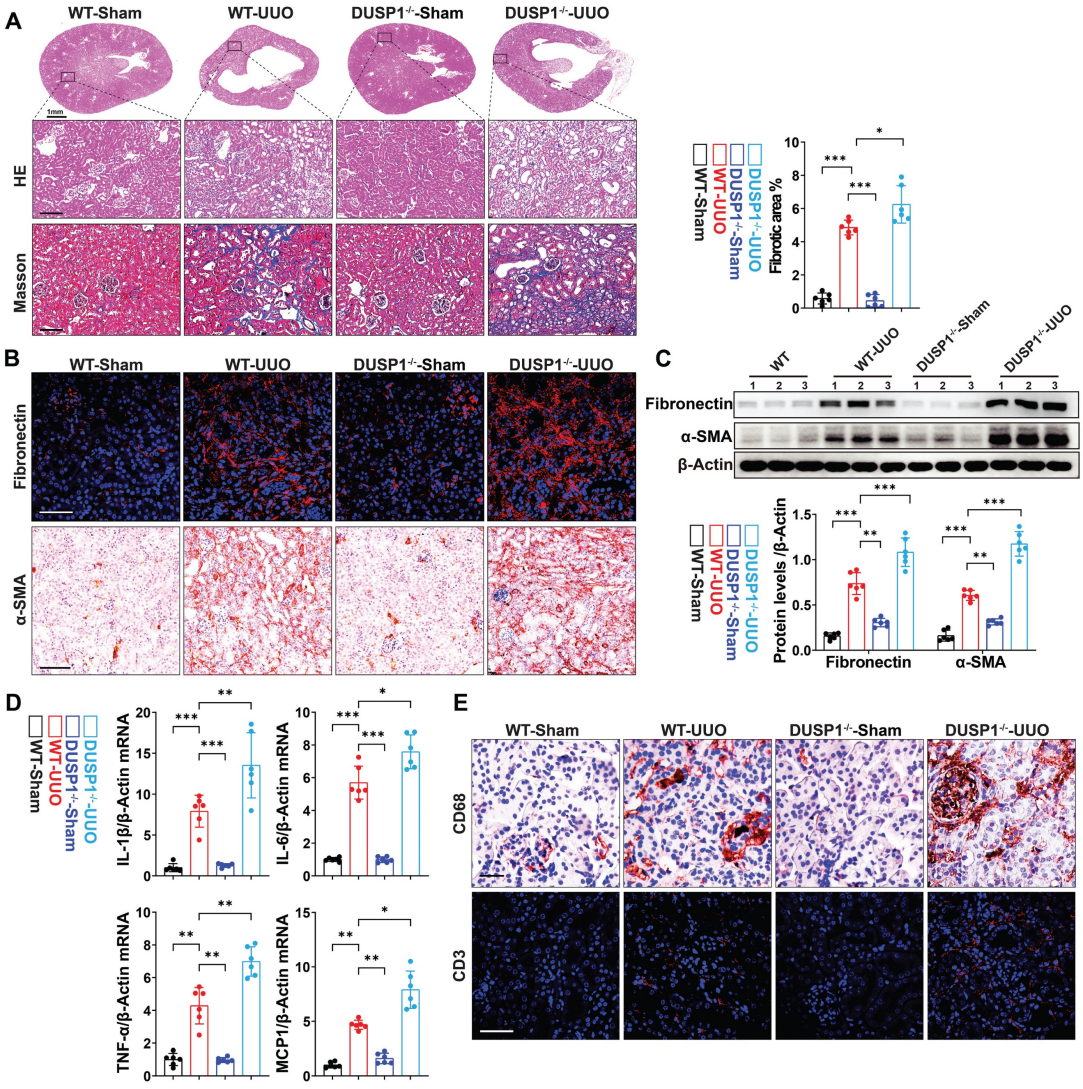
**DUSP1 deficiency accelerates UUO-induced kidney injury and fibrosis.** (A) Representative H&E and Masson staining of the kidneys of wild-type (WT) and DUSP1 knockout mice after UUO (n = 6). Scale bars, 100µm. The bar graph illustrates the quantification of renal fibrotic areas as determined by Masson staining. (B) Representative immunofluorescence and immunohistochemical images. Top row scale: 50 µm; bottom row scale: 100 µm. (C) Protein levels of fibronectin and α-SMA in the kidneys of wild-type (WT) and DUSP1 knockout mice after UUO. (D) Relative mRNA levels of IL-1β, IL6, TNF-α, and MCP-1 in the kidneys of wild-type (WT) and DUSP1 knockout mice subjected to UUO surgery. (E) Representative immunofluorescence and immunohistochemical images of CD68 and CD3. Top row scale: 10 µm; bottom row scale: 50 µm. *p < 0.05, **p < 0.01, and ***p < 0.001, n.s., not significant, one-way ANOVA with Bonferroni's post-test (A, C, D).

Subsequently, we explored the potential impact of DUSP1 deficiency on UUO-induced renal fibrosis by employing DUSP1 knockout (DUSP1^-/-^) mice generated using the CRISPR/Cas9 system (Figure S2A). Verification of DUSP1 expression in these mice confirmed the absence of DUSP1 at both mRNA and protein levels, as demonstrated by qPCR and western blot analyses (Figure S2B-D). Immunofluorescence staining further corroborated the lack of DUSP1 protein expression in the kidneys of the DUSP1^-/-^ mice (Figure S2E). Notably, DUSP1^-/-^ mice exhibited more severe renal tubular injury than wild-type mice, as evidenced by increased swelling and cavitation of RTECs and more pronounced renal fibrosis, as indicated by Masson's trichrome staining (Figure 2A). Moreover, DUSP1 knockout led to an exacerbation of profibrotic markers, such as fibronectin and α-smooth muscle actin (α-SMA), in UUO kidneys, highlighting intensified fibrotic changes in the absence of DUSP1 (Figure 2B-C). Furthermore, interstitial inflammation was exacerbated in DUSP1^-/-^ mice under UUO, evidenced by heightened infiltration of inflammatory cells and excessive production of inflammatory cytokines (Figure 2D-E, Figure S2F). These findings suggested that the absence of DUSP1 exacerbates kidney damage, enhances inflammatory infiltration, and aggravates UUO-induced fibrosis.

### DUSP1 deficiency promoted TGF-β-induced fibrogenesis and the nuclear translocation of Smad 3 in RTECs

**Figure 3:**
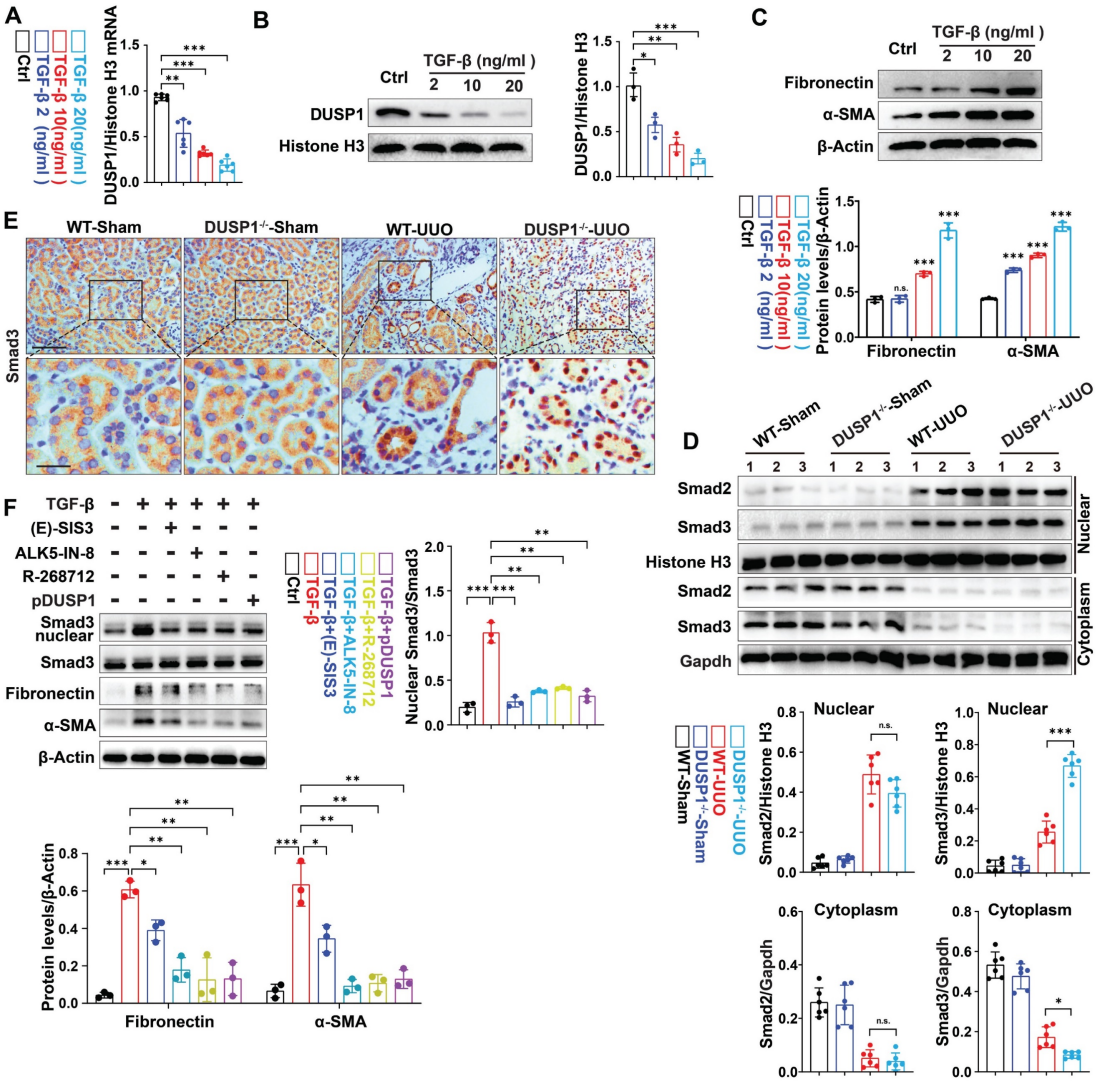
** DUSP1 deficiency promotes the nuclear translocation of Smad3 and contributes to fibrosis.** (A) Relative mRNA and (B) protein levels of DUSP1 in HK-2 cells treated with different concentrations of TGF-β. (C) Protein expression levels of fibronectin and α-SMA in HK-2 cells treated with various concentrations of TGF-β. (D) Protein expression of Smad2 and Smad3 in the kidneys of wild-type (WT) and DUSP1 knockout (DUSP1^-/-^) mice after UUO. Histone H3 and GAPDH were used as loading controls. (E) Representative immunohistochemical images of Smad3 expression in the kidneys of WT and DUSP1^-/-^ mice after UUO. Above row: scale bar = 100 μm; below row: scale bar = 20 μm. The bar charts show the results of statistical analyses of the IF assays. (F) Following treatment of HK-2 cells with TGF-β, the expression of Smad3, fibronectin, and α-SMA was assessed after administration of Smad3 inhibitors, including (E)-SIS3 (10 nM), ALK5-IN-8 (10 nM), and R-268712 (10 nM), as well as after transfection with a DUSP1 plasmid. *p < 0.05, **p < 0.01, and ***p < 0.001, n.s., not significant, one-way ANOVA with Bonferroni's post-test (A-D and F).

Next, *in vitro* experiments revealed that DUSP1 expression decreased in HK-2 cells in a dose-dependent manner after transforming growth factor-β (TGF-β) stimulation (Figure 3A-C). Therefore, we investigated the potential inhibitory effects of DUSP1 overexpression on kidney fibrosis. To this end, we introduced DUSP1 (DUSP1 overexpressed using an adenoviral vector) into HK-2 cells. Following transfection, the HK-2 cells were treated with TGF-β. As anticipated, overexpression of DUSP1 resulted in the suppression of kidney fibrosis markers (Figure S3A-B). Studies have demonstrated that DUSP1 mostly dephosphorylates active MAPKs, resulting in their inactivation through phosphate removal and subsequent termination of their cellular activity 24. DUSP1 has been validated to operate on p38, ERK, and JNK 25. Next, we observed that the deletion of DUSP1 significantly modified the phosphorylation of p38, whereas JNK and ERK remained unaffected during UUO (Figure S3C), indicating that DUSP1 may mitigate fibrosis by reducing p38 activation. However, the application of a p38 phosphorylation inhibitor did not hinder fibrogenesis in TGF-β-treated HK-2 cells (Figure S3D), suggesting that DUSP1 does not affect fibrosis via MAPK signaling. Therefore, we investigated other potential downstream pathways of DUSP1. Extensive evidence has shown that TGF-β/Smad signaling is crucial in kidney fibrosis 17. TGF-β directly triggers the activation of Smad2/3, which subsequently combines with Smad4 in the nucleus to initiate the fibrotic process 26. Given the significance of Smad3 in this process, we examined its correlation with DUSP1 expression. Intriguingly, our study revealed that DUSP1 deficiency led to an increase in nuclear Smad3 expression, whereas Smad2 levels remained unaffected (Figure 3D).

Moreover, immunohistochemical staining revealed that DUSP1 deficiency was associated with the nuclear translocation of Smad3 (Figure 3E), indicating that DUSP1 may regulate fibrosis through its interaction with Smad3. To further confirm our hypothesis, an *in vitro* experiment with siDUSP1 was conducted, which further promoted the expression of Smad3 in the nucleus under TGF-β treatment, proving that DUSP1 and Smad3 have a regulatory relationship (Figure S3E). This hypothesis was further validated by inhibiting Smad3 nuclear translocation using three different drugs in conjunction with overexpression of DUSP1. The results demonstrated a partial reversal of TGF-β-induced fibrogenesis following the intervention (Figure 3F). Collectively, these findings suggest that DUSP1 mitigates fibrosis by impeding the nuclear translocation of Smad3.

### DUSP1 dephosphorylated Smad3 mostly at 423/425 residues

**Figure 4:**
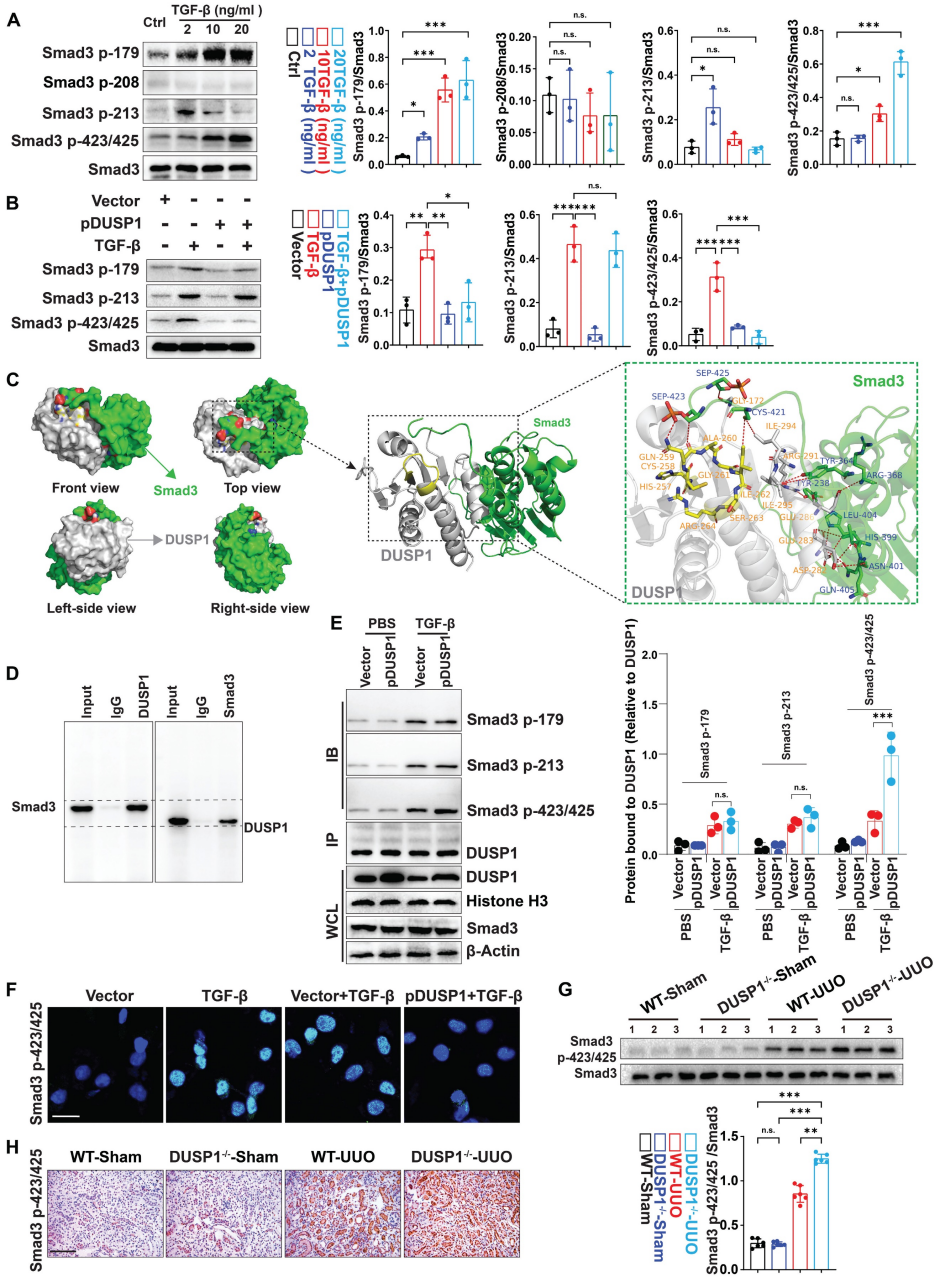
** DUSP1 dephosphorylates Smad3 mainly at residues 423/425.** (A) Protein expression of Smad3 with phosphorylation sites in HK-2 cells following treatment with various concentrations of TGF-β for 24h. (B) Protein expression of Smad3, including its phosphorylation sites, in HK-2 cells after transfection with a DUSP1-overexpressing plasmid and treatment with or without TGF-β for 24 h. (C) Molecular docking of human Smad3 and DUSP1. (D) Co-IP assay of the interaction between DUSP1 and Smad3 in HK-2 cells treated with TGF-β. (E) Co-IP assays of the interaction between DUSP1 and phosphorylation sites 179, 213, and 423/425 of Smad3. (F) Representative immunofluorescence images of Smad3 with phosphorylation sites 423/425 in HE-2 cells transfected with a DUSP1 overexpressing plasmid and treated with or without TGF-β for 24 h. Scale bars, 20µm. (G) Protein expression of Smad p423/425 in the kidneys of WT and DUSP1 knockout (DUSP1^-/-^) mice after UUO. (H) Representative immunohistochemical staining images of Smad p423/425 in the kidneys of WT and DUSP1 ^-/-^ mice after UUO. Scale bars:200 µm. *p < 0.05, **p < 0.01, and ***p < 0.001, n.s., not significant, one-way ANOVA with Bonferroni's post-test (A, B, E and G).

Phosphorylation of serine residues in Smad3 has been shown to play a role in various disorders in previous research 27. As DUSP1 is involved in MAPK dephosphorylation, it is plausible that it is also involved in Smad3 dephosphorylation. To explore this, we measured phosphorylated Smad3 (p-Smad3) levels at serine residues 179, 208, 213, and 423/425. The investigation revealed a significant dose-dependent increase in p-Smad3 levels at residues 179 and 423/425 following TGF-β stimulation (Figure 4A). However, DUSP1 overexpression resulted in reduced p-Smad3 levels at these residues (Figure 4B). Additionally, we analyzed the binding model of Smad3 and DUSP1, which revealed an interaction between the C-terminal of Smad3 and the phosphotyrosine-binding loop (PTP-loop) of DUSP1. Notably, the phosphoserine residues Sep^423^ and Sep^425^ of Smad3 formed hydrogen bonds with Gln^259^ in the PTP-loop and with Gly^172^ of DUSP1 (Figure 4C). Co-immunoprecipitation experiments further substantiated the interaction between DUSP1 and Smad3, particularly at residues 423/425 (Figure 4D-E). To better verify the interaction between Smad3 and DUSP1, we subsequently mutated cystine to valine (C258V) and glutamine to aspartic acid (Q259D) *in vitro*. The C258V-Q259D DUSP1 variant exhibited substantially lower dephosphorylation activity and only partially retained its interaction with Smad3 (Figure S4A-C). Furthermore, *in vitro* experiments confirmed that DUSP1 overexpression suppressed Smad3 phosphorylation at 423/425 (Figure 4F). In the UUO model, Smad3 and its site-specific phosphorylation at residues 423/425 were markedly upregulated in the kidneys of DUSP1^-/-^ mice (Figure 4G-H). Collectively, these findings suggest that DUSP1 plays a pivotal role in the regulation of Smad3 phosphorylation.

### DUSP1 deacetylation attenuated TGF-β induced smad3 phosphorylation and profibrotic markers in RTECs

**Figure 5:**
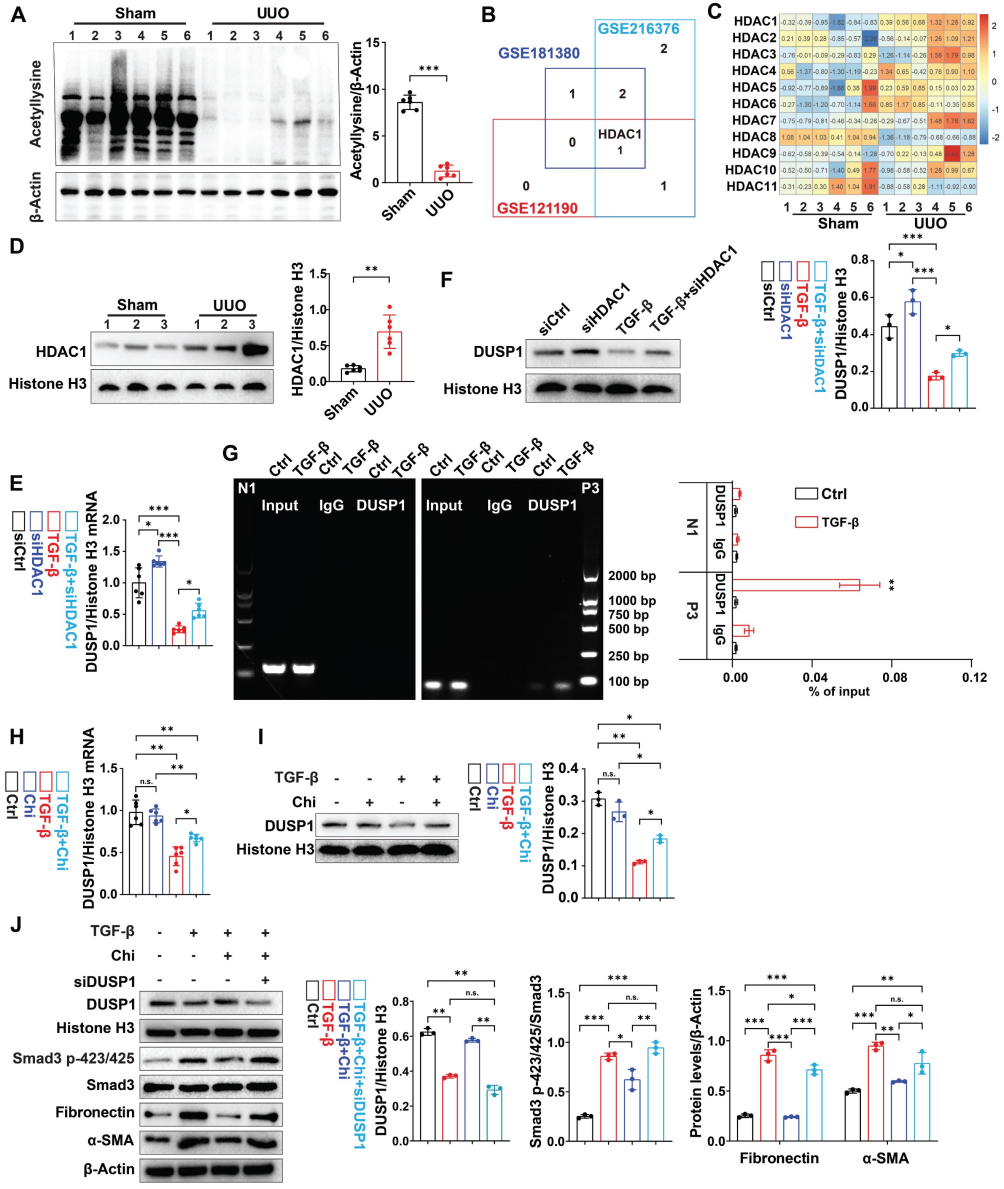
**DUSP1 is downregulated by acetylation in UUO.** (A) Western blot analysis of acetylation in the kidneys of the sham and UUO mice. (B) Venn diagram analysis was performed using the GEO RNA-seq database (GSE216376, GSE181380, and GSE121190). (C-D) Relative mRNA of HDAC family members expression of HDAC1 in the kidneys of sham and UUO mice. (E-F) Relative mRNA and protein expression levels of DUSP1 in HK-2 cells transfected with siHDAC1 in the presence or absence of TGF-β. (G) ChIP assay for DUSP1 and HDAC1 in HK-2 cells treated with control or TGF-β for 24 h. IgG was used as the negative control. Data are expressed as the percentage of input DNA. (H-I) HK-2 cells were treated for 24 h with TGF-β in the presence or absence of chidamide (Chi), and DUSP1 expression was detected by qPCR and western blotting. (J) HK-2 cells were treated for 24 h with TGF-β in the presence or absence of Chi, and DUSP1, Smad3p-423/425, smad3, fibronectin, and α-SMA were detected using western blotting. *p < 0.05, **p < 0.01, and ***p < 0.001, n.s., not significant, student t test (A), or one-way ANOVA with Bonferroni's post-test (C-J).

Next, we investigated the potential regulatory mechanisms of DUSP1 in UUO model. Using bioinformatic tools, we predicted the transcription factors for DUSP1. Of the 12 putative transcription factors examined, only activating protein-1 (AP-1) and specificity protein 1 (SP-1) showed differential expression between UUO mice and sham-operated mice (Figure S5A). Next, we determined whether DUSP1 was regulated by epigenetic modifications, including acetylation, methylation, and ubiquitination. Then, we administered acetylation inhibitor (Trichostatin A, TSA), proteasome inhibitor (Mg132), and methyltransferase inhibitor (Decitabine) to HK-2 cells along with TGF-β. Intriguingly, we found that only TSA, a class I/II HDAC inhibitor, successfully reversed the decrease in DUSP1 expression; neither Mg132 nor Decitabine treatment did (Figure S5B-C). This suggested that DUSP1 expression may be modulated by acetylation. Consequently, we observed a reduction in acetylation levels in the kidneys of UUO mice compared to those in sham mice (Figure 5A). Subsequent analysis of an online GSE database revealed significant upregulation of HDAC1 in UUO mice (Figure 5B). In the UUO model, HDAC1 mRNA expression was also significantly elevated (Figure 5C). Further examination revealed significant upregulation of HDAC1 in the kidneys of UUO mice compared to that in sham mice (Figure 5D). Moreover, our analysis demonstrated high HDAC1 expression in RTECs of human CKD biopsy samples (Figure S5D). Furthermore, an *in vitro* experiment confirmed that siHDAC1 (Figure S5E-F) inhibited the TGF-β-induced decrease in DUSP1 expression in HK-2 cells (Figure 5E-F). Notably, Chromatin Immunoprecipitation (ChIP) showed that HDAC1 was recruited to the DUSP1 promoter, supporting its regulatory role in DUSP1 expression (Figure 5G). We then tested the effect of chidamide, an HDAC1 inhibitor, on DUSP1 and fibrosis-related indicators *in vitro*. Surprisingly, chidamide increased DUSP1 expression, decreased fibrosis markers and hindered the phosphorylation of Smad3 (Figure 5H-J). These findings suggest that HDAC1-induced epigenetic modifications may control DUSP1 expression and fibrosis in UUO mice.

### Histone deacetylase inhibitor alleviated the downregulation of DUSP1 expression and renal fibrosis in the kidney of UUO mice

**Figure 6:**
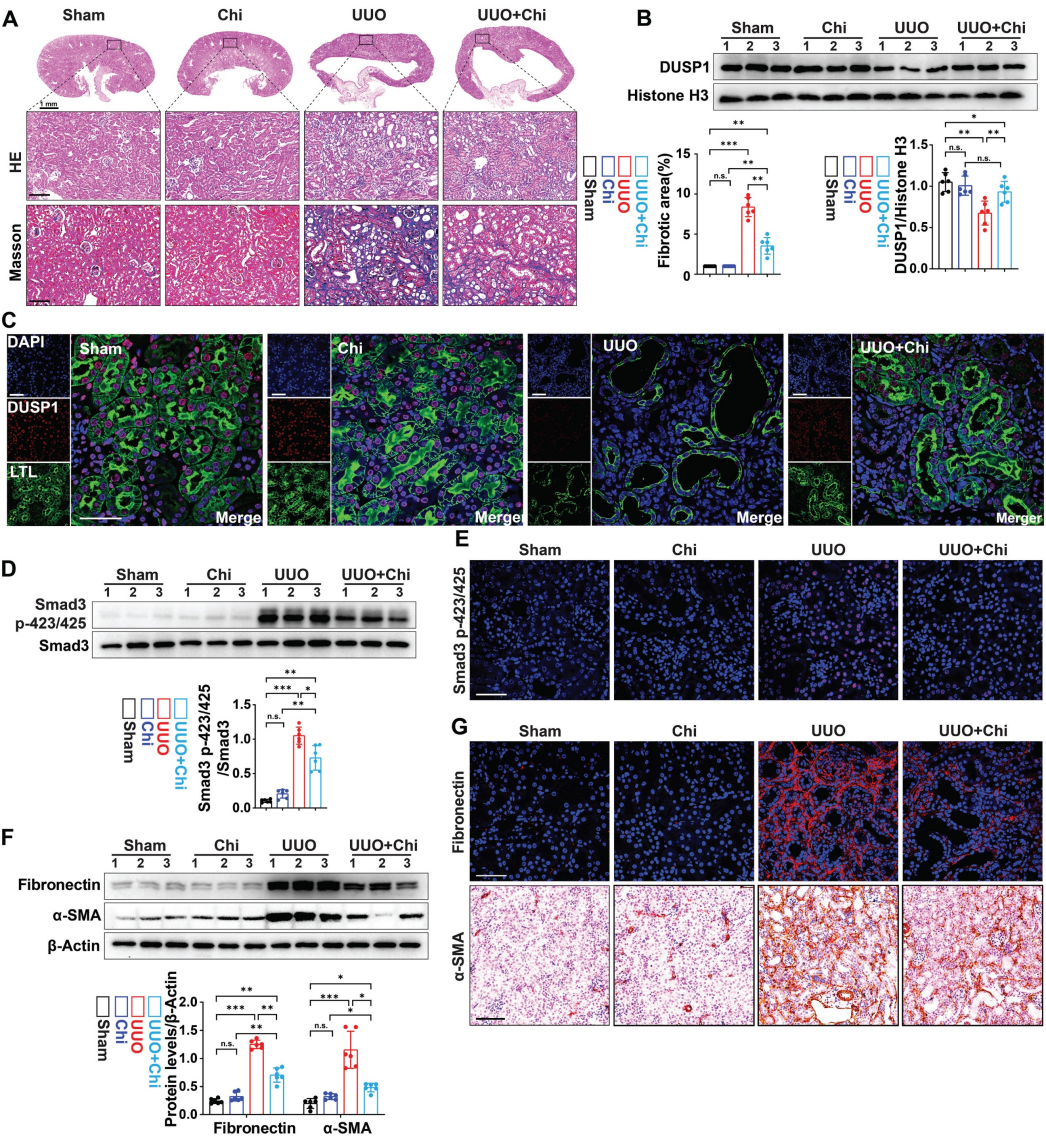
** HDAC1 inhibition is involved in the development of renal fibrosis.** (A) Representative H&E and Masson staining of the kidneys of sham and UUO mice pre-treated with or without Chi. Scale bars, 100 µm. (B) Protein expression levels of DUSP1 in the kidneys of sham and UUO mice pre-treated with or without Chi. (C) Representative immunofluorescence images and quantification analysis of DUSP1. Scale bars, 50 μm. (D) Protein levels of Smad3 and Smad3 p423/425 in the kidneys of sham and UUO mice pre-treated with or without Chi. (E) Representative immunofluorescence images of Smad3 p423/425 in the kidneys of sham and UUO mice pre-treated with or without Chi. Scale bars: 50 µm. (F) Protein levels of fibronectin and α-SMA in the kidneys of sham and UUO mice pre-treated with or without Chi. (G) Representative immunofluorescence images and quantification analysis of fibronectin and α-SMA levels in the kidneys of sham and UUO mice pre-treated with or without Chi. Top row scale: 50 µm; bottom row scale: 100 µm. *p < 0.05, **p < 0.01, and ***p < 0.001, n.s., not significant, one-way ANOVA with Bonferroni's post-test (A, B, D and F).

Subsequently, the therapeutic efficacy of chidamide was further examined by administering chidamide to mice prior to UUO. Compared to the UUO group, chidamide-treated mice showed reduced tubular damage and fibrosis in the renal tissue (Figure 6A). Moreover, western blot analysis and immunofluorescence revealed that the expression of DUSP1 was restored in UUO mice treated with chidamide, in contrast to mice that were not treated with chidamide (Figure 6B-C). In addition, chidamide inhibited the activation of Smad3 and renal fibrosis in the kidneys of UUO mice (Figure 6D-G). These findings indicate that chidamide has a significant potential as a therapeutic agent for renal fibrosis.

### DUSP1 overexpression attenuated UUO-induced renal fibrosis

**Figure 7:**
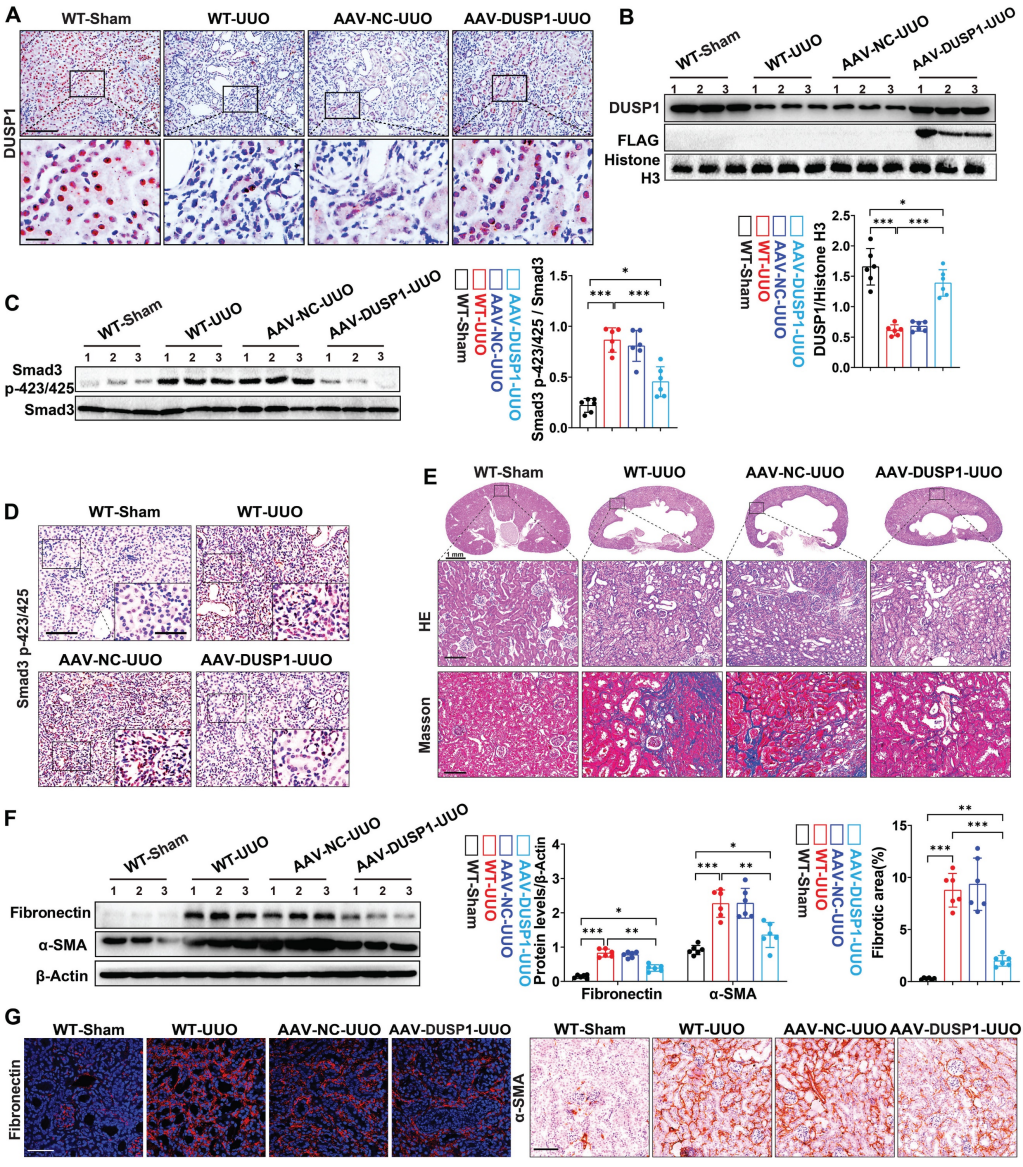
**DUSP1 overexpression attenuates UUO-induced renal injury and fibrosis.** (A) Representative immunohistochemical staining images of DUSP1 in kidneys of sham and UUO mice injected with or without AAV-DUSP1. Above row: scale bar = 100 μm; below row: scale bar = 20 μm. Quantitative analysis of DUSP1 expression (n = 6). (B) Protein expression levels of DUSP1 and FLAG in the kidneys of sham and UUO mice injected with or without AAV-DUSP1. (C) Protein expression of Smad3 p423/425 in the kidneys of sham and UUO mice injected with or without AAV-DUSP1. (D) Representative immunohistochemical staining images of Smad3 p423/425 in kidneys of sham and UUO mice injected with or without AAV-DUSP1. Scale bars: 100 µm for full view and 10 µm for partially enlarged view. (E) Representative H&E and Masson staining of the kidneys of sham and UUO mice injected with or without AAV-DUSP1. Scale bars, 100µm. (F) Protein expression of fibronectin and α-SMA in the kidneys of sham and UUO mice injected with or without AAV-DUSP1. (G) Representative immunofluorescence and immunohistochemical images of fibronectin and α-SMA in kidneys of sham and UUO mice injected with or without AAV-DUSP1. Left panels: Scale bars, 50 µm; Right panels: Scale bars, 100 µm. *p < 0.05, **p < 0.01, and ***p < 0.001, n.s., not significant, one-way ANOVA with Bonferroni's post-test (B, C, E and F).

To further validate the renoprotective role of DUSP1 *in vivo*, we conducted an overexpression experiment using AAV vectors in a UUO model (Figure S6A-C). Successful transduction of DUSP1 was confirmed by anti-flag immunofluorescence labeling (Figure S6D). AAV-DUSP1 or its empty control was administered via tail injection immediately after UUO surgery. Our observations indicated that DUSP1 levels were significantly elevated in the renal tubules of AAV-DUSP1-transfected mice compared to those of NC mice (Figure 7A). Western blot analysis confirmed that DUSP1 expression in the AAV-DUSP1 group was restored to levels similar to those in the sham group (Figure 7B). DUSP1 overexpression not only inhibited smad3 phosphorylation (Figure 7C-D) but also alleviated kidney injury and fibrosis (Figure 7E-F). Furthermore, Immunohistochemical staining and immunofluorescence revealed notable attenuation of elevated fibronectin and α-SMA levels in the AAV-DUSP1 group (Figure 7G). These results unequivocally demonstrated that DUSP1 overexpression can effectively alleviate kidney fibrosis and may represent a possible translational potential of a promising therapeutic strategy.

## Discussion

**Figure 8:**
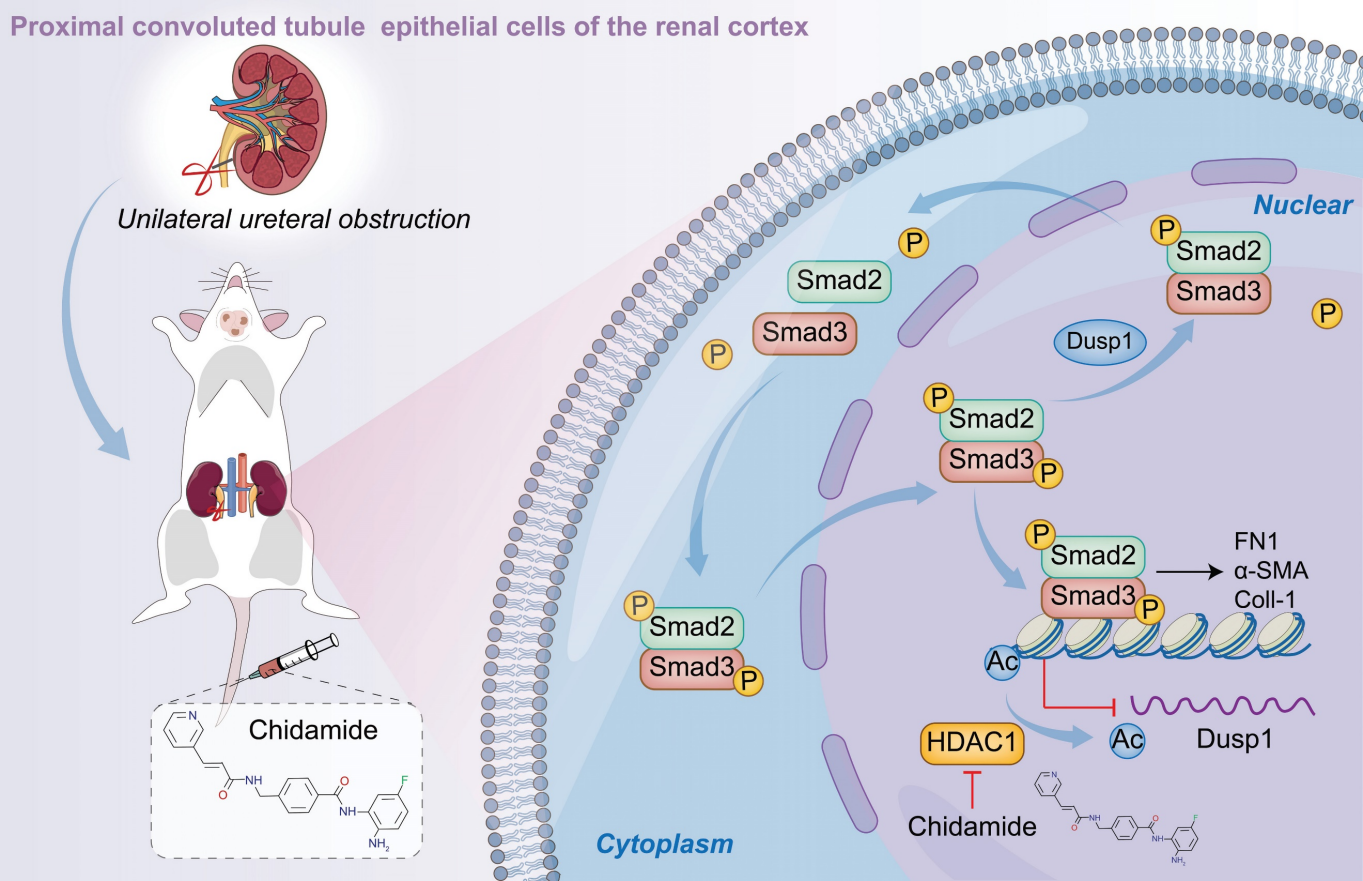
Schematic representation of the hypothesized role of DUSP1 in modulation of renal fibrosis. Under UUO conditions, the downregulation of DUSP1 is predominantly governed by acetylation, which impedes the dephosphorylation of Smad3, particularly at residues 423/425. Phosphorylated Smad3 directly facilitates renal fibrosis. In contrast, overexpression of DUSP1 or use of an HDAC1 inhibitor can impede renal fibrosis.

We investigated the pivotal role of DUSP1 in the progression of kidney fibrosis in UUO model and TGF-β-stimulated human kidney tubular epithelial cells. Additionally, we assessed DUSP1 expression in kidney tissues of patients with CKD. The overexpression of DUSP1 effectively mitigates kidney fibrosis both *in vitro* and *in vivo*. Moreover, we identified DUSP1 as a potential regulator of TGF-β-induced fibroblast activation via direct dephosphorylation of Smad3 at residues 423/425. Finally, we demonstrated that the downregulation of DUSP1 is modulated by HDAC1. Silencing or inhibition of HDAC1 restored DUSP1 expression and alleviated kidney fibrosis (Figure 8).

DUSP1 plays a crucial role in regulating cell proliferation, differentiation, and survival. It is involved in various diseases including cancer, inflammation, and neurodegenerative disorders 28. Recent studies have shown that DUSP1 expression is significantly reduced in patients with CKD, which is often accompanied by kidney dysfunction and fibrosis 29. A previous study also showed that DUSP1 serves as a sensitive biomarker for the diagnosis of focal segmental glomerular sclerosis and diabetic kidney disease (DKD) 30, 31. Lower DUSP1 expression was associated with renal dysfunction and glomerular apoptosis, while overexpression of DUSP1 interrupted Mff-related mitochondrial fission, reducing hyperglycemia-mediated mitochondrial damage and thus improving renal function 32. In addition, DUSP1 overexpression ameliorated inflammatory markers related to MAP kinase pathways in human tubular epithelial cells 33. DUSP1 overexpression in HK-2 cells partially restored autophagic flux, improved mitochondrial function, and reduced reactive oxygen species generation and cell apoptosis under high-glucose conditions 34. These studies indicate that DUSP1 plays a crucial role in regulating kidney function and preventing fibrosis. Using single-cell sequencing, we found that DUSP1 was mostly expressed in renal tubular cells. We demonstrated that DUSP1 is significantly downregulated in various CKD and fibrosis models. DUSP1 is associated with kidney dysfunction and fibrosis in patients with CKD. Thus, DUSP1 may be a potential therapeutic target for renal fibrosis. We discovered that DUSP1 knockout promoted kidney injury and fibrosis caused by UUO, whereas overexpression of DUSP1 inhibited TGF-β-induced fibrotic effects and attenuated UUO-induced kidney fibrosis. These findings could be important for the development of new therapies for renal fibrosis.

Previous studies have shown that DUSP1 protects against kidney injury and fibrosis mainly through its classic signaling pathways, including p38, ERK, and JNK, which impair their cellular functions. For example, DUSP1 protects against acute kidney injury by stabilizing mtDNA through interactions with JNK 35. DUSP1 attenuates HG-induced expression of collagen I, collagen IV, and fibronectin via inactivation of the p38 MAPK and ERK1/2 pathways 36. It is significantly different from previous studies, our study found that DUSP1 directly inhibits kidney injury and fibrosis by regulating Smad3, rather than its classical signaling pathway. For the first time, we demonstrated that DUSP1 deficiency facilitates the nuclear translocation of Smad3 and contributes to the development of kidney fibrosis. Smad3 is an important signal transmitter within cells for TGF-β signaling 37 and can interact with multiple signaling pathways to mediate renal inflammation and fibrosis 38. Thus, targeting Smad3 attenuates fibrosis, apoptosis, and inflammation 39. Previous studies have shown that Smad3 is specifically dephosphorylated by protein phosphatase 2A specifically under hypoxic conditions 40. Overexpression of MAN1 leads to the dephosphorylation of Smad2 and Smad3 41, RanBP3 directly recognizes dephosphorylated Smad2/3 42, also PPM1A functions as a Smad phosphatase to terminate TGF-β signaling 43. DUSP1 inhibits activin/Smad2-mediated organizer gene expression and FGF8-mediated neural induction 44. In our study, we found that DUSP1 overexpression inhibited Smad3 phosphorylation both *in vitro* and *in vivo*; however, the exact regulatory mechanism remains unclear. Smad3 regulates downstream signaling, principally through phosphorylation. We further explored specific phosphorylation sites. Using network molecular precipitation and co-immunoprecipitation experiments, we confirmed that DUSP1 dephosphorylates Smad3 at residues 423/425. These findings provide new insights into the mechanism by which DUSP1 regulates the development of fibrosis.

Interestingly, we also found that DUSP1 was downregulated by acetylation, whereas HDAC1 was highly expressed under UUO conditions. Knockdown or inhibition of HDAC1 reversed the expression of DUSP1 and alleviated renal fibrosis. This suggests that HDAC1 may be a potential therapeutic target for the treatment of CKD. Research indicates that aldosterone induces renal fibrosis by promoting HDAC1 expression, deacetylating H3K9, and inhibiting klotho transcription 45. Inhibition of HDAC enhances STAT acetylation, blocks NF-κB, and suppresses renal inflammation and fibrosis in Npr1 haplotype male mice 46. HDAC1 inhibition by MS-275 in mesothelial cells limits cellular invasion and promotes MMT reversal 47. Our study showed that HDAC1 is highly expressed in kidney fibrosis. At the same time, the HDAC1 inhibitor chidamide, which is also an anti-tumor drug used for T-cell lymphoma and other hematological malignancies, is being studied 48, 49. Chidamide is being studied as a potential HDAC inhibitor for the treatment of renal fibrosis through various mechanisms 50, 51, including the regulation of profibrotic genes, the induction of apoptosis to eliminate fibrous tissue 52, and the inhibition of the epithelial-to-mesenchymal transition (EMT) pathway to prevent fibroblast formation 53. Overall, chidamide demonstrates significant potential for mitigating renal fibrosis and may be suitable for therapeutic application. Further clinical trials are required to validate these findings.

## Conclusions

In conclusion, our findings highlight the critical involvement of DUSP1 in kidney function and fibrosis, suggesting that targeting DUSP1 or HDAC1 may represent a promising therapeutic approach for renal fibrosis. Further investigations are warranted to comprehensively elucidate the mechanisms through which DUSP1 modulates these processes and to develop efficacious therapies based on these findings.

## Supplementary Material

Supplementary figures and tables.

## Abbreviations

CKD: Chronic kidney disease
DUSP1: Dual specificity phosphatase 1
MAPK: Mitogen-activated protein kinase
UUO: Unilateral ureteral obstruction
RTECs: Renal tubular epithelial cells
ECM: Extracellular matrix proteins
HDAC1: Histone deacetylase 1
eGFR: Estimated glomerular filtration rate
BUN: Blood Urea Nitrogen
CRE: Serum Creatinine
α-SMA: α-smooth muscle actin
TGF-β: Transforming growth factor-β
PTP-loop: Phosphotyrosine binding loop
AP-1: Activating protein-1
SP-1: Specificity protein 1
TSA: Trichostatin A
Chi: Chidamide
