# Supplementary material for: Acetylation-regulated DUSP1 deficiency contributes to renal fibrosis progression
Source: Theranostics. 2025 Mar 3;15(9):3781–96. doi: 10.7150/thno.108992 (PMC11980654; doi:10.7150/thno.108992)
Supplement: Supplementary file 1 — Supplementary figures and tables. [file thnov15p3781s1.pdf]

Supplemental Information

Acetylation-regulated DUSP1 deficiency contributes to renal fibrosis progression

Shaobo Wang[1], Bo Zhang[1], Yaqin Wang[1], Qigang Lan[1], Liangjing Lv[1], Tangli Xiao[1], Yan Li[1], Mengying Yao[1], Jun Zhang[1], Cheng Wang[2], Yinghui Huang[1], Jinghong Zhao[1]*, Jiachuan Xiong[1]*

[1] Department of Nephrology, the Key Laboratory for the Prevention and Treatment of Kidney Disease of Chongqing, Chongqing Clinical Research Center of Kidney and Urology Diseases, Xinqiao Hospital, Army Medical University, Third Military Medical University), Chongqing, 400037, China.

[2] State Key Laboratory of Trauma, Burns and Combined Injury, Institute of Combined Injury, Chongqing Engineering Research Center for Nanomedicine, College of Preventive Medicine, Army Medical University (Third Military Medical University), Chongqing, China.

*** Corresponding Authors:** Jiachuan Xiong, Department of Nephrology, the Key Laboratory for the Prevention and Treatment of Kidney Disease of Chongqing, Chongqing Clinical Research Center of Kidney and Urology Diseases, Xinqiao Hospital, Army Medical University, E-mail: xiongjc@tmmu.edu.cn; ORCID: 0000-0003-2430-3711

*** Corresponding Authors:** Jinghong Zhao, Department of Nephrology, the Key Laboratory for the Prevention and Treatment of Kidney Disease of Chongqing, Chongqing Clinical Research Center of Kidney and Urology Diseases, Xinqiao Hospital, Army Medical University, Third Military Medical University), Chongqing, 400037, China. E-mail: zhaojh@tmmu.edu.cn; ORCID: 0000-0001-9750-3285

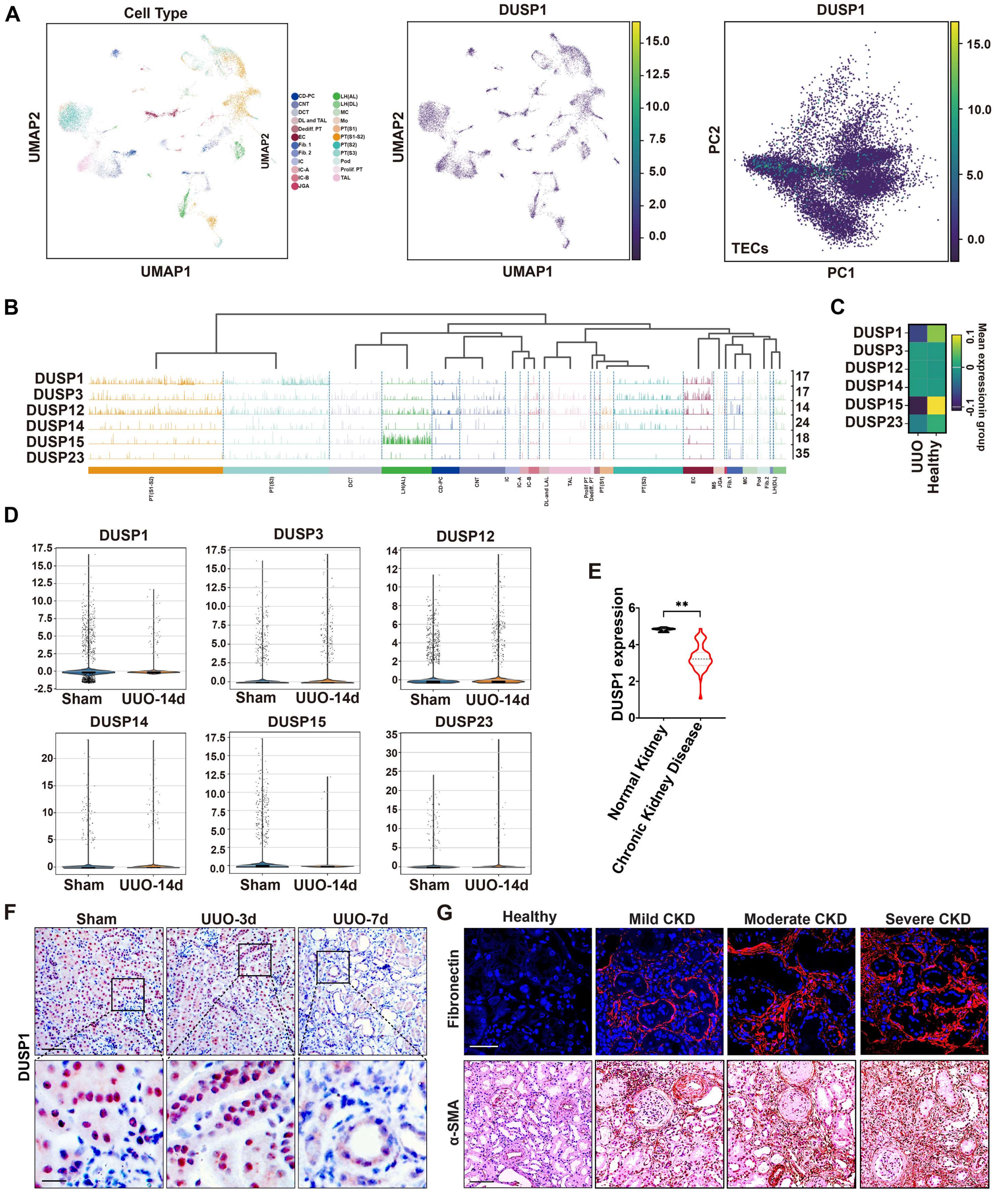

Figure S1. Single-cell sequence analysis of DUSP1 expression using an online database. (A) DUSP1 expression in different cell groups using the UMAP algorithm. (B-C) DUSP1, DUSP3, DUSP12, DUSP14, DUSP15, and DUSP23 expression in UUO mice and sham groups. (D) RNA-

seq analysis of DUSP1, DUSP3, DUSP12, DUSP14, DUSP15, and DUSP23 expression levels using an online database. (E) RNA-seq analysis of DUSP1 expression in chronic kidney disease using an online database. (F) Representative immunohistochemistry images of DUSP1 expression levels on days 3 and 7 in the kidneys of the sham and UUO mice. (G) Representative immunofluorescence and immunohistochemical images of kidney tissues from patients at various stages of CKD. Scale bars, 50 μm. *P < 0.05 and **P < 0.01. ***P < 0.001. n.s., not significant. Data were analyzed using an unpaired Student's t-test and expressed as the mean ± SD. Data are representative of at least three independent experiments.

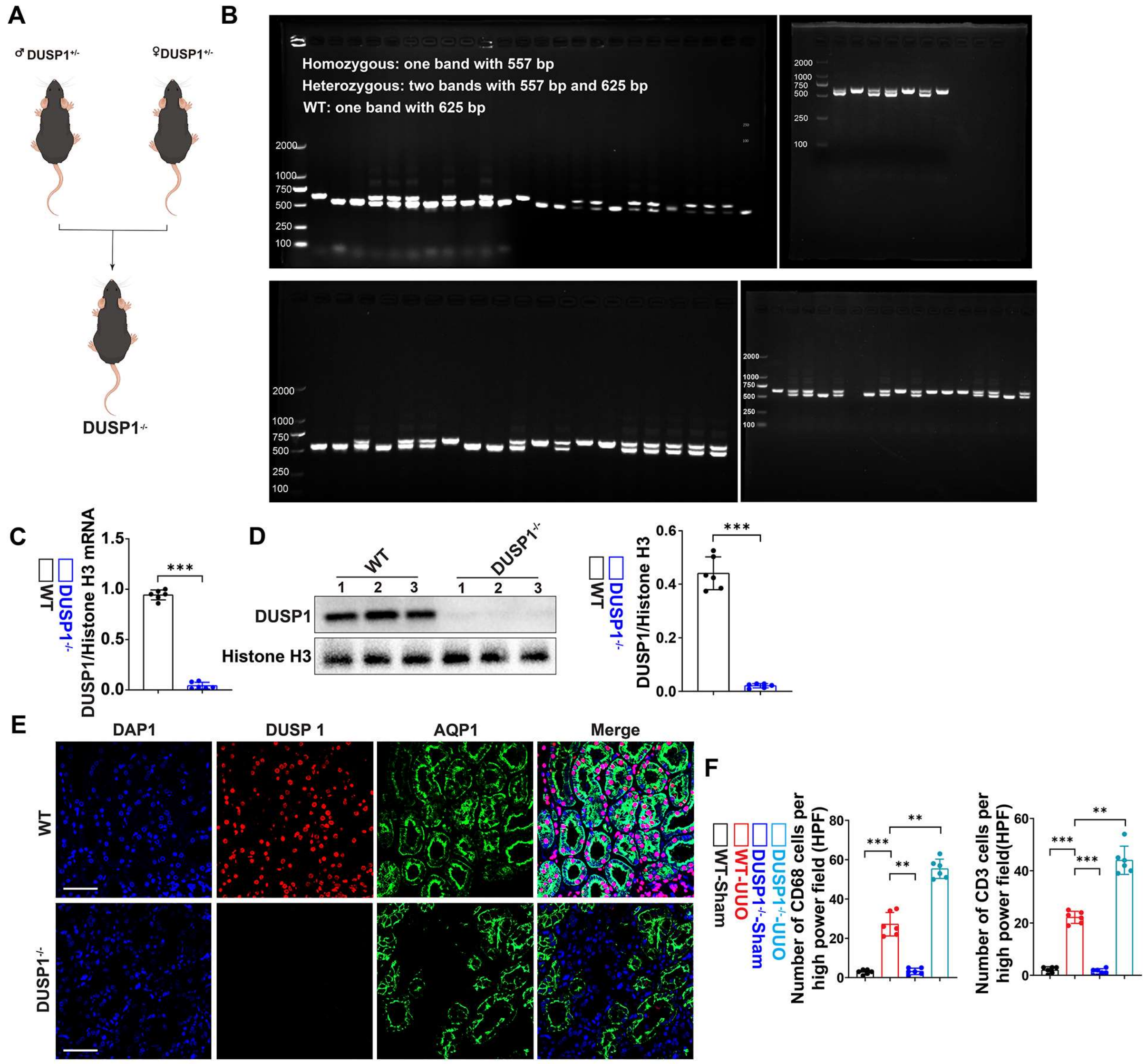

Figure S2. Identification of DUSP1 gene knockout. (A-B) DUSP1-KO mice were developed using the CRISPR/Cas9 system. The DNA extracted from the tails of mice was subjected to PCR and agarose gel electrophoresis, and the following results were obtained: the DNA band of DUSP1$^{-/-}$ mice was approximately 557 bp, and the DNA band of WT mice was approximately 625 bp. The hybrid mice had two bands (557 bp and 625 bactin as the loading control. (E) Representative immunofluorescence images of DUSP1 expression. Scale bars, 50 μm. (F) The fraction of CD68 and CD3 positive cells was analyzed across the WT-Sham, Dusp1-Sham, WT-UUO, and Dusp1-

UUO groups. Scale bars, 50 μm. * $P < 0.05$ and ** $P < 0.01$. *** $P < 0.001$. n.s., not significant. Data were analyzed using an unpaired Student's t-test and are shown as mean ± SD. Data are representative of at least three independent experiments.

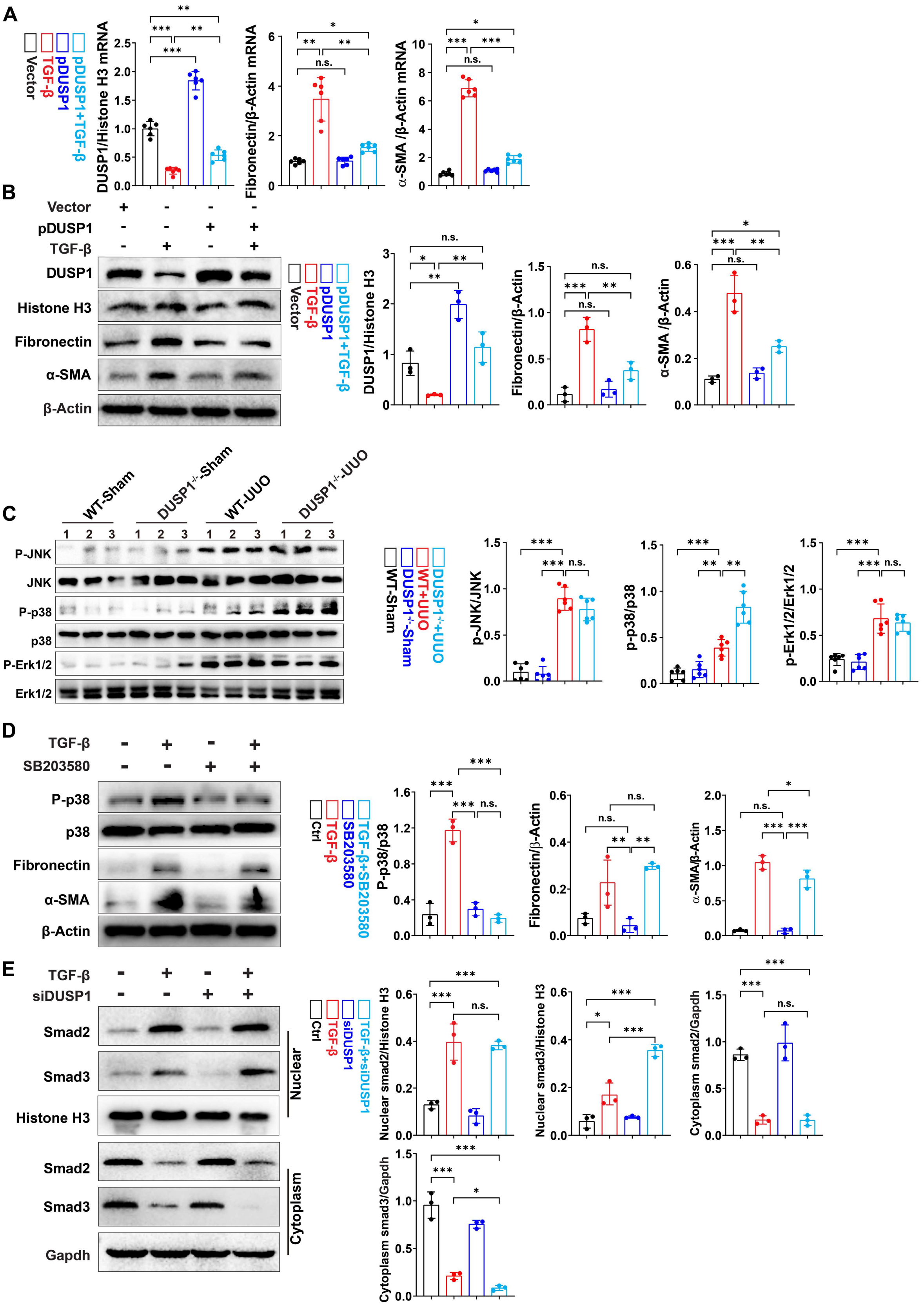
A
Vector
TGF-β
pDUSP1
pDUSP1+TGF-β
DUSP1/Histone H3 mRNA
Fibronectin/β-Actin mRNA
α-SMA /β-Actin mRNA
n.s.
B
Vector
pDUSP1
TGF-β
DUSP1
Histone H3
Fibronectin
α-SMA
β-Actin
DUSP1/Histone H3
Fibronectin/β-Actin
α-SMA /β-Actin
C
WT-Sham
DUSP1-/--Sham
WT-UUO
DUSP1-/--UUO
P-JNK
JNK
P-p38
p38
P-Erk1/2
Erk1/2
WT+UUO
DUSP1-/-+UUO
p-JNK/JNK
p-p38/p38
p-Erk1/2/Erk1/2
D
TGF-β
SB203580
P-p38
p38
Fibronectin
α-SMA
β-Actin
Ctrl
TGF-β+SB203580
P-p38/p38
Fibronectin/β-Actin
α-SMA/β-Actin
E
TGF-β
siDUSP1
Smad2
Smad3
Histone H3
Nuclear
Cytoplasm
Gapdh
TGF-β+siDUSP1
Nuclear smad2/Histone H3
Nuclear smad3/Histone H3
Cytoplasm smad2/Gapdh
Cytoplasm smad3/Gapdh

Figure S3. Impact of overexpression of DUSP1 on α-SMA and fibronectin expression in HK-2 cells under TGF-β treatment. (A) mRNA expression of DUSP1, fibronectin, and α-SMA in HK-2 cells, with and without DUSP1 overexpression, in the presence or absence of TGF-β treatment. (B) Western blot analysis of DUSP1, fibronectin, and α-SMA expression in HK-2 cells with and without DUSP1 overexpression in the presence or absence of TGF-β treatment. (C) Western blot analysis of MAPK phosphorylated or non-phosphorylated expression in WT-Sham, Dusp1-Sham, WT-UUO, DUSP1-UUO, β-actin as the loading control (n=6). Protein expression of phosphorylated or non-phosphorylated MAPK in the kidneys of WT and DUSP1 knockout ($DUSP1^{-/-}$) mice after UUO. (D) Western blot analysis of p-p38, p38, fibronectin, and α-Sma expressions in HK-2 cells treated with SB203580 inhibitor (10 μM) or in the presence or absence of TGF-β. (E) Representative immunoblots showing nuclear and cytoplasmic fractions of Smad2 and Smad3 in HK-2 cells treated with TGF-β or subjected to siRNA-mediated DUSP1 silencing. * $P < 0.05$ and ** $P < 0.01$. *** $P < 0.001$. n.s., not significant. Data were analyzed using an unpaired Student's t-test and are shown as mean ± SD. Data are representative of at least three independent experiments.

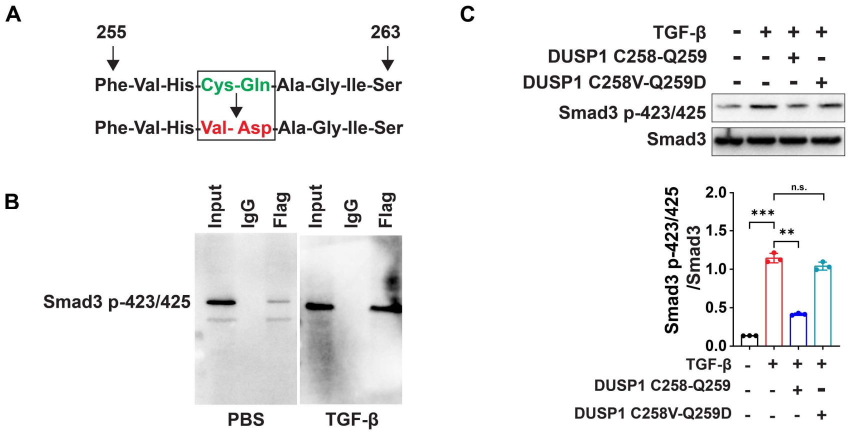

Figure S4. Effect of Cys^{258} and Gln^{259} mutations in DUSP1 on its interaction with Smad3. (A) Mutations in cystine to valine (C258V) and glutamine to aspartic acid (Q259D) were introduced into DUSP1. (B) Co-IP assay evaluating the interaction between mutant FLAG-DUSP1 C258V/Q259D and HIS-Smad3 p-423/425 in HEK293T cells. (C) Western blot analysis was performed to evaluate the interaction between mutant DUSP1 (C258V/Q259D) and phosphorylated Smad3 at Ser423/425 in HEK293T cells. * $P < 0.05$ and ** $P < 0.01$. *** $P< 0.001$. n.s., not significant. Data were analyzed by an unpaired Student's t-test and shown as means ± SD. Data are representative of at least three independent experiments.

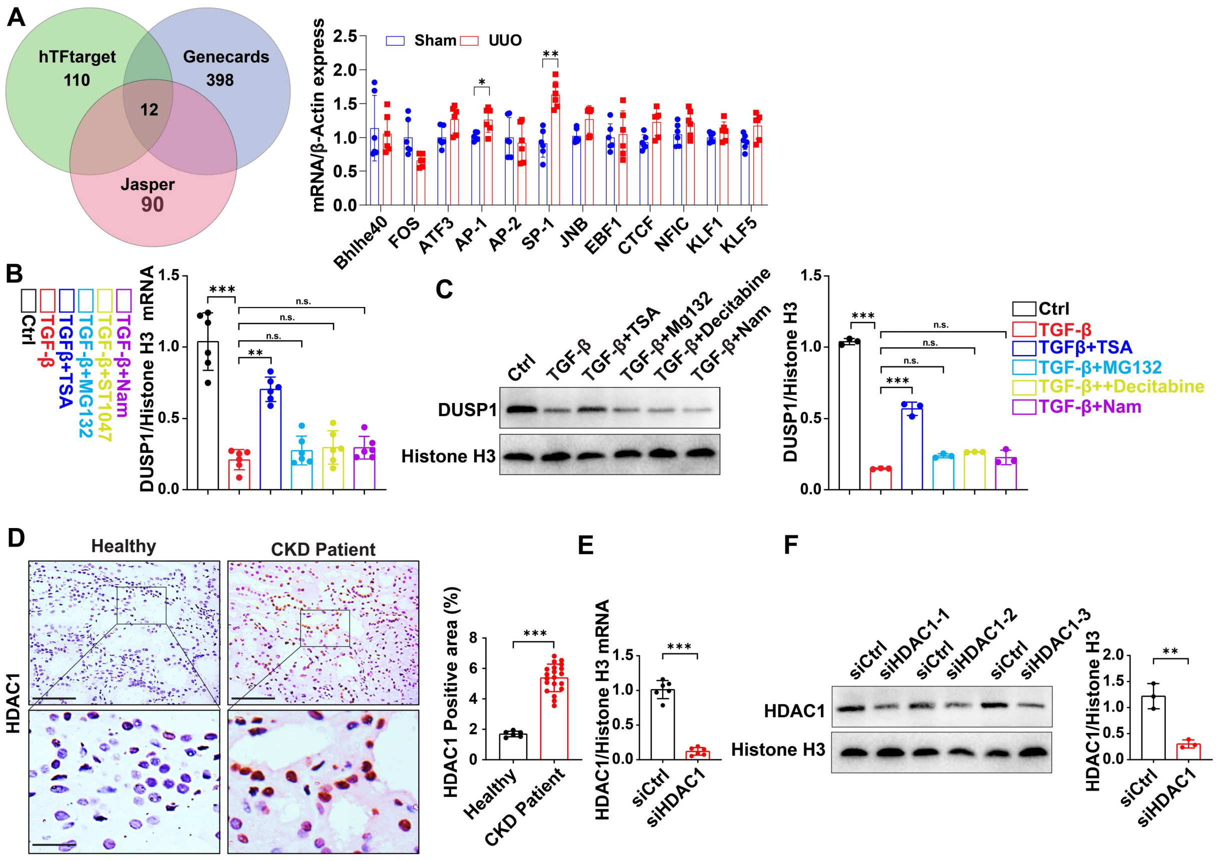

Figure S5. Analysis of DUSP1 regulation and HDAC1 Expression in HK-2 Cells and CKD patients. (A) Transcriptional factors in the DUSP1 gene promoter region were predicted using JASPAR, hTFtarget, and GeneCards (gene-regulation) software programs, and 12 common transcription factors were identified. (B-C) DUSP1 expression was examined in HK-2 cells treated with methylation inhibitors (10 nM), acetylation inhibitors (10 nM), or ubiquitination inhibitors (10 nM) in the presence or absence of TGF-β, using qPCR and western blot analysis. (D) HDAC1 staining images of kidney biopsies from healthy controls and patients with CKD. Above row: scale bar = 200 μm; below row: scale bar = 20 μm. (E-F) Verification of the effect on HDAC1 mRNA expression using qPCR and western blot analysis after transfection with siHDAC1-1, siHDAC1-2, and siHDAC1-3. * P < 0.05 and ** P < 0.01. *** P< 0.001. n.s., not significant. Data were

analyzed using an unpaired Student's t-test and are shown as mean ± SD. Data are representative of at least three independent experiments.

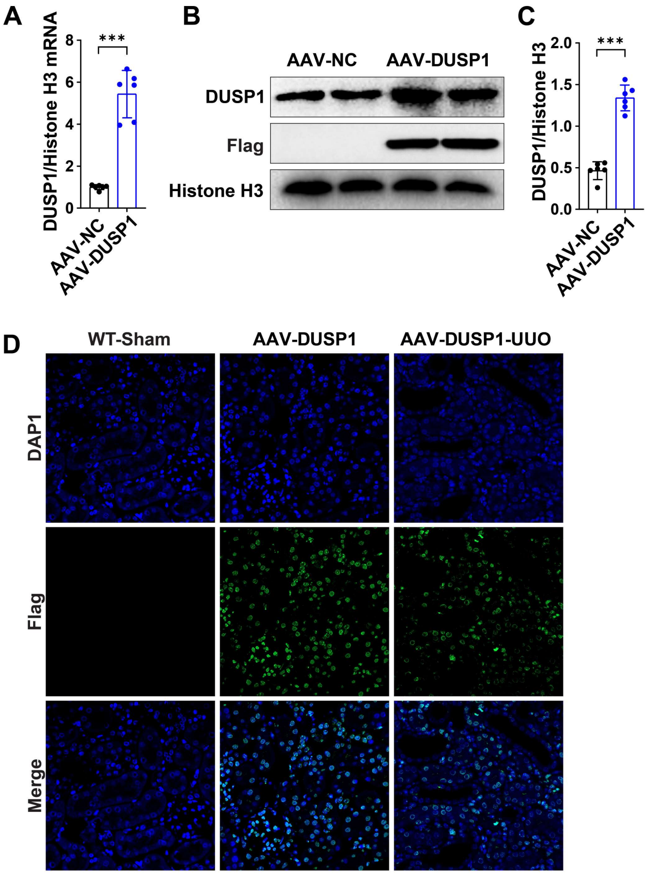

Figure S6. Validation of renal DUSP1 expression after AAV-DUSP1-Flag injection. (A-C) Validation of DUSP1 mRNA (A) and protein (B-C) expression in the kidney following intravenous injection of AAV-DUSP1-Flag using qPCR and western blot analysis. (D) Representative immunofluorescence images of Flag (green). * $P < 0.05$ and ** $P < 0.01$. *** $P < 0.001$. Data were analyzed using an unpaired Student's t-test and are shown as mean ± SD. Data are representative of at least three independent experiments.

Table S1 The basic characteristic of the included CKD patients

Patients No.	Sex	Age (years)	Diagnosis	serum creatine μmol/L	BUN mmol/L	eGFR (mL/min1.73 m^2)	Urine protein quantitation (g/24h)	Cystatin C (mg/L)
1	Male	49	CKD	504.50	20.46	11	9.09	3.87
2	Male	64	CKD	574.40	26.18	8	NA	3.96
3	Female	47	CKD	311.00	12.91	15	4.02	2.98
4	Male	33	CKD	338.60	14.76	19	3.95	2.91
5	Male	44	CKD	171.60	8.67	41	0.93	1.84
6	Male	68	CKD	127.30	9.14	50	24.64	1.24
7	Male	67	CKD	299.40	13.87	18	7.32	2.73
8	Male	68	CKD	443.70	20.12	11	0.35	3.43
9	Female	42	CKD	213.90	9.95	24	1.61	2.05
10	Female	51	CKD	189.50	16.52	26	0.18	2.67
11	Female	35	CKD	119.70	7.33	51	0.78	1.51
12	Male	58	CKD	185.80	9.18	34	0.93	2.32
13	Male	47	CKD	121.40	8.41	61	1.2	1.54
14	Male	65	CKD	212.50	11.58	27	NA	2.43
15	Female	68	CKD	143.60	9.48	32	3.12	1.8
16	Male	58	CKD	143.50	15.72	46	10.41	1.87
17	Female	42	CKD	369.90	12.37	12	2.28	3.58
18	Male	28	CKD	259.90	11.77	28	3.48	3.26
19	Female	56	CKD	580.30	11.86	6	NA	10.3
20	Male	55	CKD	111.70	6.57	64	2.56	1.42
21	Female	30	CKD	187.70	19.11	36	NA	1.5

Table S2. The siRNA sequences are listed in table below.

Name	Position	Target sequence	RNA oligo sequences
siDUSP1-1	105-127	GTCCAAAAGCGGCTTTTGGTTCG	AACCAAAAGCCGCUUUUGGAC<br>CCAAAAGCGGCUUUUGGUUCG
siDUSP1-2	767-789	TGGAAATCCTGCCCTTTCTGTAC	ACAGAAAGGGCAGGAUUUCCA<br>GAAAUCCUGCCCUUUCUGUAC
siDUSP1-3	864-886	AGCCAATTGTCCCAACCATTTTG	AAAUGGUUGGGACAAUUGGCU<br>CCAAUUGUCCCAACCAUUUUG
siHdac1-1	229-251	TACCGAAAAATGGAAATCTATCG	AUAGAUUUCCAUUUUUCGGUA<br>CCGAAAAAUGGAAAUCUAUCG
siHdac1-2	111-133	GAGGAAAGTCTGTTACTACTACG	UAGUAGUAACAGACUUUCCUC<br>GGAAAGUCUGUUACUACUACG
siHdac1-3	136-158	GGGGATGTTGGAAATTACTATTA	AUAGUAAUUUCCAACAUCCCC<br>GGAUGUUGGAAAUUACUAUUA

Table S3. Primer sequences are listed in the table below

Species	Name		Sequence (5'->3')	Length	Amplicon Size
human	DUSP1	Forward primer	GCCTTGCTTACCTTATGAGGAC	22	86
		Reverse primer	GGGAGAGATGATGCTTCGCC	20	
	Fibronectin	Forward primer	GAGGGCCAAGACGAAGACATC	21	140
		Reverse primer	CAGATCACGTCATCGCACAAC	21	
	α-SMA	Forward Primer	AAAAGACAGCTACGTGGGTGA	21	76
		Reverse Primer	GCCATGTTCTATCGGGTACTTC	22	
	HDAC1	Forward Primer	CGCCCTCACAAAGCCAATG	19	108
		Reverse Primer	CTGCTTGCTGTACTCCGACA	20	
	β-Actin	Forward primer	CACTGTCGAGTCGCGTCC	18	89
		Reverse primer	TCATCCATGGCGAACTGGTG	20	
Mouse	DUSP1	Forward Primer	AGCTGTGCAGCAAACAGTCCA	21	258
		Reverse Primer	ACTGGTAGTGACCCTCAAAGTGG	23	
	HDAC1	Forward Primer	CTACTACGACGGGGATGTTGG	21	77
		Reverse Primer	GAGTCATGCGGATTCGGTGAG	21	
	HDAC2	Forward Primer	ATGGCGTACAGTCAAGGAGG	20	112
		Reverse Primer	TGCGGATTCTATGAGGCTTCA	21	
	HDAC3	Forward Primer	CCTGGCATTGACCCATAGCC	20	168
		Reverse Primer	CTCTTGGTGAAGCCTTGCATA	21	
	HDAC4	Forward Primer	GGCCCACCGGAATCTGAAC	19	87
		Reverse Primer	GAACTCTGGTCAAGGGAACTG	21	
	HDAC5	Forward Primer	TCTTGTCGAAGTCAAAGGAGC	21	108
		Reverse Primer	GAGGGGAACTCTGGTCCAAAG	21	
	HDAC6	Forward Primer	AAGAAGACCTAATCGTGGGACT	22	248
		Reverse Primer	GCTGTGAACCAACATCAGCTC	21	
	HDAC7	Forward Primer	GGCGGCCCTAGAAAGAACAG	20	205
		Reverse Primer	CTTGGGCTTATAGCGCAGCTT	21	
	HDAC8	Forward Primer	TCGCTGGTCCCGGTTTATATC	21	82
		Reverse Primer	TACTGGCCCGTTTGGGGAT	19	
	HDAC9	Forward Primer	AGTAGAGAGGCATCGCAGAGA	21	141
		Reverse Primer	GGAGTGTCTTTCGTTGCTGAT	21	
	HDAC10	Forward Primer	CAGTTCGACGCCATCTACTTC	21	115
		Reverse Primer	CAAGCCCATTTTGCACAGCTC	21	
	HDAC11	Forward Primer	ACCCAGACAGGAGGAACCATA	21	130
		Reverse Primer	TGATGTCCGCATAGGCACAG	20	
	Fibronectin	Forward Primer	GGCCACCATTACTGGTCTGG	20	132
		Reverse Primer	GGAAGGGTAACCAGTTGGGG	20	
	α-SMA	Forward Primer	CCCTGGAGAAGAGCTACGAAC	21	204
		Reverse Primer	TACCCCCTGACAGGACGTTG	20	
	IL-1β	Forward Primer	GAAATGCCACCTTTTGACAGTG	22	116
		Reverse Primer	TGGATGCTCTCATCAGGACAG	21	
	IL-6	Forward Primer	CTGCAAGAGACTTCCATCCAG	21	131
		Reverse Primer	AGTGGTATAGACAGGTCTGTTGG	23	

TNF-α	Forward Primer	ACCCTCACACTCACAAACCA	20	212
	Reverse Primer	ATAGCAAATCGGCTGACGGT	20	
MCP-1	Forward Primer	CACTCACCTGCTGCTACTCA	20	117
	Reverse Primer	GCTTGGTGACAAAAACTACAGC	22	
β-Actin	Forward primer	TCCCATTGAACACGGAGTCG	20	116
	Reverse primer	CCTCGGTGAGAAGAATAGGATGT	23	

Table S4. Antibody information is listed in the table below

Antibody name	Article No.	Production company	Country
Anti-DUSP1	#48625	CST	USA
Anti-DUSP1	AF6750	Beyotime Biotechnology	China
Anti-DDDDK tag	ab205606	Abcam	USA
Anti-Beta Actin	ab8226	Abcam	USA
Anti-Smad2	ab40855	Abcam	USA
Anti-Smad3	ab40854	Abcam	USA
Anti-Smad3 (phospho S213)	ab63403	Abcam	USA
Anti-Smad3 (phospho S423 + S425)	ab118825	Abcam	USA
Anti-Smad3 (phospho T179)	ab74062	Abcam	USA
Anti-Smad3 (phospho S208)	ab138659	Abcam	USA
Anti-Histone H3	AF0009	Beyotime Biotechnology	China
Anti-GAPDH	AF0006	Beyotime Biotechnology	China
Anti-Fibronectin	ab2413	Abcam	USA
Anti-alpha smooth muscle Actin	ab7817	Abcam	USA
Anti-HDAC1	ab109411	Abcam	USA
Anti-Acetyllysine	PTM-101	PTMBIO	China
Anti-CD3	#86603	CST	USA
Anti-CD68	#97778	CST	USA

Table S5. Chip primer sequences are listed in the table below

Species	Name		Sequence (5'->3')	Length	Amplicon Size
Human	P1	Forward primer	TGAAAAATTTCGGTAGGGGAAGG	23	94
		Reverse primer	AGCTGCTGACTTTTCACTGC	20	
	P2	Forward primer	ATGCAGTGAAAAGTCAGCAGC	21	73
		Reverse primer	ATTCAGCTGAGGAACTCAACA	21	
	P3	Forward Primer	AAAATTTCGGTAGGGGAAGGT	21	90
		Reverse Primer	GCTGCTGACTTTTCACTGCAT	21	
	N1	Forward Primer	TCCATGTGATGGCATGTGGT	20	118
		Reverse Primer	GCGGCTGGTTACTTGAGAGT	20	
	N2	Forward Primer	AATGGAATGGATGCCTGGGG	20	130
		Reverse Primer	TGCCCACACCAGACTGATAC	20	
	N3	Forward primer	TGGCATGTGGTGATGTTGAC	20	110
		Reverse primer	AGCGGCTGGTTACTTGAGAG	20	
